# Chemokines in depression in health and in inflammatory illness: a systematic review and meta-analysis

**DOI:** 10.1038/mp.2017.205

**Published:** 2017-11-14

**Authors:** S P Leighton, L Nerurkar, R Krishnadas, C Johnman, G J Graham, J Cavanagh

**Affiliations:** 1 Institute of Health and Wellbeing, University of Glasgow, Glasgow, UK; 2 Institute of Infection, Immunity and Inflammation, University of Glasgow, Glasgow, UK; 3 Institute of Neuroscience and Psychology, University of Glasgow, Glasgow, UK; 4 Institute of Health and Wellbeing, College of Medical, Veterinary and Life Sciences, University of Glasgow, Glasgow, UK

## Abstract

Inflammatory illness is associated with depression. Preclinical work has shown that chemokines are linked with peripheral–central crosstalk and may be important in mediating depressive behaviours. We sought to establish what evidence exists that differences in blood or cerebrospinal fluid chemokine concentration discriminate between individuals with depression and those without. Following PRISMA guidelines, we systematically searched Embase, PsycINFO and Medline databases. We included participants with physical illness for subgroup analysis, and excluded participants with comorbid psychiatric diagnoses. Seventy-three studies met the inclusion criteria for the meta-analysis. Individuals with depression had higher levels of blood CXCL4 and CXCL7 and lower levels of blood CCL4. Sensitivity analysis of studies with only physically healthy participants identified higher blood levels of CCL2, CCL3, CCL11, CXCL7 and CXCL8 and lower blood levels of CCL4. All other chemokines examined did not reveal significant differences (blood CCL5, CCL7, CXCL9, CXCL10 and cerebrospinal fluid CXCL8 and CXCL10). Analysis of the clinical utility of the effect size of plasma CXCL8 in healthy individuals found a negative predictive value 93.5%, given the population prevalence of depression of 10%. Overall, our meta-analysis finds evidence linking abnormalities of blood chemokines with depression in humans. Furthermore, we have demonstrated the possibility of classifying individuals with depression based on their inflammatory biomarker profile. Future research should explore putative mechanisms underlying this association, attempt to replicate existing findings in larger populations and aim to develop new diagnostic and therapeutic strategies.

## Introduction

Depressive disorders are the third leading cause of years lost to disability worldwide, rising to second among 15–49 year olds. This burden has increased over the past 25 years.^1^ Our current understanding of depression and its diagnosis and treatment centres around the monoamine hypothesis. Yet, 30% of patients fail to respond to such anti-depressants and among responders only a third attain remission.^2^ This high rate of treatment failure likely reflects an incomplete understanding of the pathogenesis of depressive disorders. Efforts are focused on effective stratification of a heterogeneous population and novel therapeutic avenues.

Over the past quarter century, an increasingly compelling body of evidence has emerged linking inflammation to depression.^3, 4, 5, 6, 7, 8, 9, 10^ Broadly, this evidence stems from four main observations:

1. A third of those with depression show elevated inflammatory cytokines, in the absence of medical illness.
2. Inflammatory illnesses are associated with greater rates of depression than noninflammatory illnesses.
3. Up to 40% of patients treated with cytokine therapy develop clinical depression.
4. Higher baseline inflammation is associated with a lack of response to anti-depressant treatment.

Preclinical work has identified *chemo*tactic cyto*kines* (chemokines) as an important group of molecules in both the immune system, where they act to coordinate immune cells and attract them to sites of ongoing inflammation, and the nervous system. Evidence indicates they play a role in neuron–glia communication, synaptic transmission, neurogenesis, neurodevelopment and plasticity and this was recently extensively reviewed by Stuart *et al.*^11^ Because of their role in cellular migration and immune coordination, chemokines are prime candidates for linking peripheral and central inflammation and orchestrating neuroinflammatory crosstalk. D’Mello *et al.*^12^ and Wohleb *et al.*^13^ highlighted the role for chemokines, particularly CCL2, in these processes in their studies of monocytic migration. Despite being widely expressed in the noninflamed adult brain,^14^ it is only in recent years that their role in neuropsychiatric disease has been explored more extensively. Recent studies have implicated CCL2, CCL3 and CXCL10 in modifications of neuronal transmission and alterations in cognitive function,^15, 16, 17^ and the neuromodulatory functions of other chemokines have been reviewed elsewhere.^18, 19^ Several chemokines, including CXCL8 and CXCL10, have been associated with alterations in neuroendocrine regulation and hypothalamic–pituitary–adrenal axis function.^20, 21^ The CXCL12–CXCR4 axis has been repeatedly identified as having a role in neurogenesis.^22, 23, 24^ Finally, crosstalk between CX3CR1 and CX3CL1 appears to be a critical pathway for communication between microglia and neurons during both physiological and pathological states.^24, 25^ Alterations in neurotransmitter and hypothalamic–pituitary–adrenal axis function and reductions in neurogenesis are consistently associated with depressive pathologies.^26, 27, 28, 29^ As such, these data provide compelling evidence of a possible role for the chemokine molecular family in the pathogenesis of depression.

Existing biomarker studies of chemokines in depression are often underpowered, of disparate methodologies and are frequently conflicting. Chemokine nomenclature is notoriously difficult despite standardisation into a unified format. Ensuring a complete coverage, especially of older literature, can be challenging. Eyre *et al.*^30^ recently undertook a meta-analysis in the field and encountered such issues that limited their interpretation to only two of the most commonly studied chemokines, CCL2 and CXCL8.

Encouragingly, even within the past few months the field continues to grow. However, the issues encountered by Eyre *et al.*^30^ remain. Aiming to be pragmatic, clinically relevant and allowing for the current state of the field, we set out to be as comprehensive as possible while still using high-quality meta-analytical techniques. We primarily achieved this aim through extensive search terms utilising as many possible variant chemokine names as possible.

In this paper, we lay out the results of our meta-analysis and frame them within the context of the current animal and human literature on inflammatory disease and depression, providing evidence for the role of chemokines in depression and identifying gaps in the literature to inform future research.

Our primary outcome:

- Do differences in blood or cerebrospinal fluid (CSF) chemokine concentration discriminate between those with and those without depression?

Our secondary outcomes:

- Do differences in blood or CSF chemokine concentration discriminate between those with and those without depression only in a healthy subgroup, or only in a subgroup with physical illness?
- Are differences in blood chemokine concentration more apparent in plasma or serum?
- Are higher-quality studies more likely to identify subgroup differences?

## Materials and methods

### Data sources

We (SPL and LN) searched Embase, PsycINFO and Medline up to 5 March 2016. We manually checked references cited in the systematically searched articles. In an effort to avoid publication bias we included non-English language studies and grey literature (for example, conference abstracts). We used a broad but highly structured search strategy based around the PICOS framework, our Population is humans, the Intervention/Exposure is depression, the Comparison is no depression, the Outcome is change in blood or CSF chemokines, and the Study design included any type of study design. Keywords for the search included various combinations of terms for depression, depressive disorders and depression research scales, and both historical and current chemokine names. Extensive use of wildcards, truncation and Boolean operators was implemented to allow for variant names (for example, CCL2 alternate search terms (monocyte chemotactic protein#1 or monocyte chemotactic protein-1 or mcp#1 or mcp-1 or small inducible cytokine a2 or gdcf#2 or gdcf-2 or hc11 or hsmcr30 or mcaf or cmc#cf or smc-cf). The full search strategy is available in the Supplementary Materials.

### Study selection

Studies were selected for data extraction and analysis based on the following inclusion criteria: (1) original research studies measuring blood or CSF chemokine concentrations in depressed and nondepressed subjects; (2) subjects met depression criteria based on a conventional psychiatric classification system or recognised depression rating scale (see search strategy); (3) control subjects did not meet said criteria for depression; (4) in an attempt to be pragmatic and increase real-world applicability, we included studies with participants suffering from physical comorbidity (any comorbidity with an established inflammatory component or sequelae) if the comorbidity was comparable and identifiable between cases and controls to allow for subgroup analysis (see characteristics of included studies table).

We excluded studies based on the following criteria: (1) participants had a comorbid or additional psychiatric diagnosis that would exclude or confound depression as per conventional psychiatric nosological thinking (that is, mania, bipolar disorder, schizophrenia or other nondepressive affective or nonaffective psychosis); (2) *in vitro* and nonhuman studies; (3) studies looking at stimulated levels of cytokines as they reflect the consequences of immune challenge as opposed to basal immune activity; (4) studies including participants who had not yet reached puberty.

### Data extraction

Two independent reviewers (SPL and LN) used a custom data extraction template adapted from the Cochrane checklist of items (see Supplementary Materials). Non-English language studies were translated. For meta-analysis, mean (±s.d.) chemokine concentrations for each group of depressed and nondepressed control subjects were extracted. We also sought data that were missing from the original reports through correspondence with the investigators. When correspondence was not successful, alternate techniques were used for data extraction. If data were presented as median (interquartile range), we used an established approximation method to estimate mean (±s.d.);^31^ if data were presented only in graphical format, we used Engauge data extraction software;^32^ if presented as raw data with chemokine concentration and depression rating scale score, participants were dichotomised based on established cutoff scores for ‘caseness’ for the relevant rating scale and mean(±s.d.) calculated; if different severities of depression were presented separately, means and s.d. were combined based on subgroup number. Such calculations were carried out within Microsoft Excel 2013. Disagreements regarding inclusion were settled by consensus.

### Quality assessment

This meta-analysis was carried out according to Preferred Reporting Items for Systematic Reviews and Meta-Analyses (PRISMA) guidelines. To assess quality we adapted the Newcastle–Ottawa Quality Assessment Scale for observational studies as recommended by the Cochrane Collaboration, together with the Cochrane common classification scheme for bias, with additional points for adjusting or matching important confounders identified from the literature (smoking, circadian rhythm (time of blood draw), body mass index, age, sex and medications), whether the source was a peer-reviewed article or an abstract, and whether we used Engauge data extraction software or data approximation giving a total score out of 17 (Supplementary Table 1). Given the small number of studies in the field, we included all accepted studies in our meta-analysis but where possible conducted sensitivity analysis only including studies that scored greater than or equal to the median quality score (⩾12/17), considered to be a low risk of bias.

### Statistical analysis

We used RevMan 5.3 (ref. ^33^) for analysis. To be conservative, we used weighted standardised mean differences (SMD) between controls and cases to calculate the effect size of each study given the heterogeneity of the assays used to measure the chemokines (for example, enzyme-linked immunosorbent assay, microarray) and, for blood chemokines, that of the modality in which they were measured (for example, serum, plasma). Weighted SMD and 95% confidence intervals (CIs) were then calculated using a random effects inverse variance model. This meta-analytic method includes both within-study variance and between-studies variation in the estimate of the uncertainty of results. Unlike a fixed effects model, a random effects model assumes that the underlying true effects vary from one study to another. A random effects model is more conservative and is chosen if significant heterogeneity is expected.

We measured statistical heterogeneity using the *I*^2^ statistic for statistical variation across studies—values of 25% are low, 50% moderate and 75% high. The presence of significant heterogeneity suggests variability in the characteristics of the trials. Likely sources of heterogeneity including presence or absence of physical illness among participants, modality of blood chemokine measurement, sex, possible outliers and quality score were investigated by sensitivity analysis where data allowed.

Additional sensitivity analyses were performed for chemokines that attained statistical significance to further explore heterogeneity. Where numbers allowed, we assessed: only studies that used a Diagnostic and Statistical Manual of Mental Disorders (DSM) Major Depressive Disorder (MDD) diagnosis, specific subtypes of illness, only articles, removing Fontenelle 2012^34^ where subjects had comorbid obsessive–compulsive disorder, removing studies where alternative methods for data extraction were required and only studies that matched for specific confounders (age, gender, body mass index, smoking, circadian rhythm of blood sampling and medication).

The possibility of publication bias was tested by visual inspection of funnel plots and by testing for asymmetry using the Egger weighted regression test within R^35^ using the metafor package.^36^ The nonparametric (rank-based) trim and fill data augmentation technique was used within R using metafor to estimate the number of studies missing from a meta-analysis because of the suppression of the most extreme results on one side of the funnel plot. The method then augments the observed data so that the funnel plot is more symmetric and its effect on the results was assessed.

To establish whether the effect sizes found in the meta-analysis had clinical utility, we examined the classification accuracy of CXCL8 in robustly discriminating cases from controls. This was possible because of the large numbers of participants eligible for inclusion, the equal distribution of cases and controls and the significant SMD with narrow confidence intervals in the plasma CXCL8 meta-analysis of healthy participants. We simulated CXCL8 data for *n* cases and *n* controls in R based on the SMD (Hegde’s *g*) derived from meta-analysis of plasma CXCL8 concentration in participants without comorbid physical illness, together with the number of cases and of controls, based on a known mean and s.d. of plasma CXCL8 in a normal healthy population (mean: 6.911 pg ml^−1^; s.d.: ±3.818 pg ml^−1^) given the assumption of a normal distribution as stated by the central limit theorem using the code outlined by Fried and Kievit^37^ (Supplementary Figure 1).

For the analysis, we estimated the classification accuracy using a logistic regression classifier with a 10-fold cross-validation procedure using Weka 3 Data Mining Software.^38^ In 10 (*n*) fold cross-validation, data are divided into 10 folds. A logistic classifier is built using *n*-1 folds, leaving out one fold. The built classifier is then tested on the left-out fold, and the classification accuracy is calculated. The above procedure is repeated *n* times by leaving out one-fold each time. Finally, the cross-validation classification accuracy is the average accuracy across all the left-out folds. In addition, we report the sensitivity, specificity, positive predictive value (PPV) and negative predictive value (NPV) of CXCL8. Here, sensitivity measures the proportion of positives that are correctly identified as such; in this case the percentage of patients who are correctly identified as having depression. Specificity measures the proportion of negatives that are correctly identified, or the percentage of healthy controls who are correctly identified as controls. PPV represents the proportion of individuals with depression among all those who tested positive, and NPV represents the proportion of healthy controls among those who tested negative. The PPV and NPV are sensitive to the prevalence of the disease. These were calculated with a conservative estimate of 10% prevalence of MDD in the general population. These quantities are computed as follows:


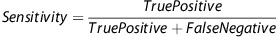



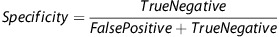



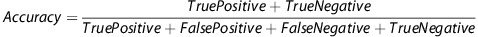



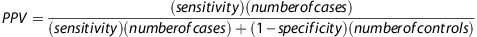



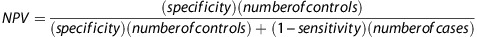


## Results

### Study inclusion

**Figure 1:**
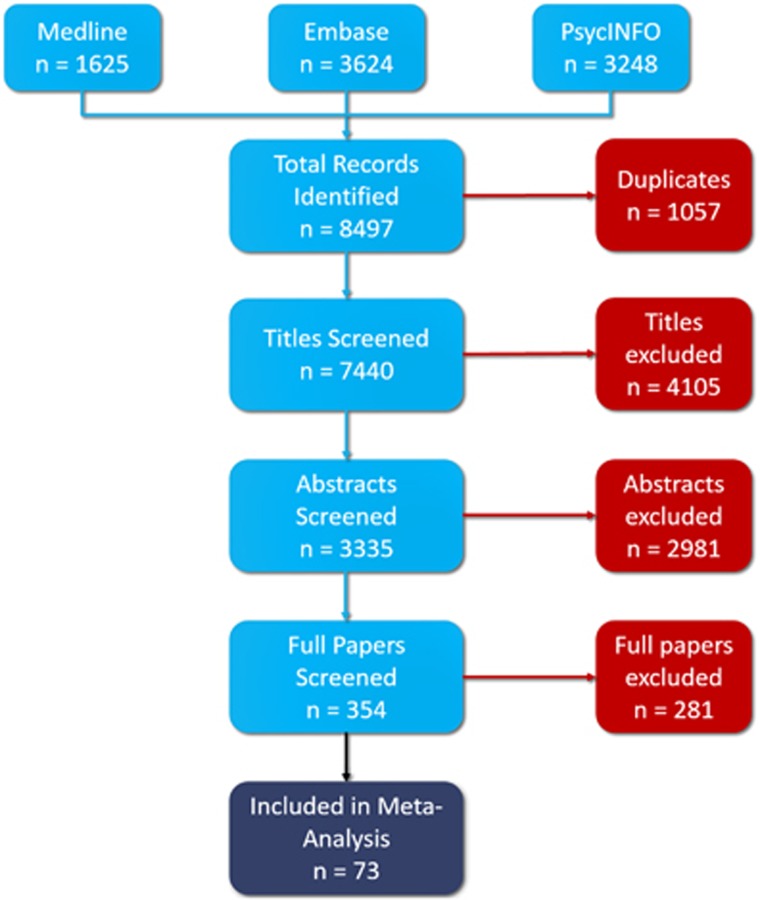
PRISMA (Preferred Reporting Items for Systematic Reviews and Meta-Analyses) flowchart showing study selection process and number of studies from each database.

The utilisation of the PRISMA guidelines and a systematic search of electronic databases yielded a total of 8497 studies. No additional studies were identified through manual searching of references. After removal of duplicates, 7440 titles were reviewed of which 4105 were excluded. Of the 3335 abstracts reviewed, 2981 were excluded. In all, 354 full texts were reviewed of which 73 met criteria for inclusion in our meta-analysis^34, 39, 40, 41, 42, 43, 44, 45, 46, 47, 48, 49, 50, 51, 52, 53, 54, 55, 56, 57, 58, 59, 60, 61, 62, 63, 64, 65, 66, 67, 68, 69, 70, 71, 72, 73, 74, 75, 76, 77, 78, 79, 80, 81, 82, 83, 84, 85, 86, 87, 88, 89, 90, 91, 92, 93, 94, 95, 96, 97, 98, 99, 100, 101, 102, 103, 104, 105, 106, 107, 108, 109^ (Figure 1 and Supplementary Table 2).

### Specific chemokines

#### CCL2

**Figure 2:**
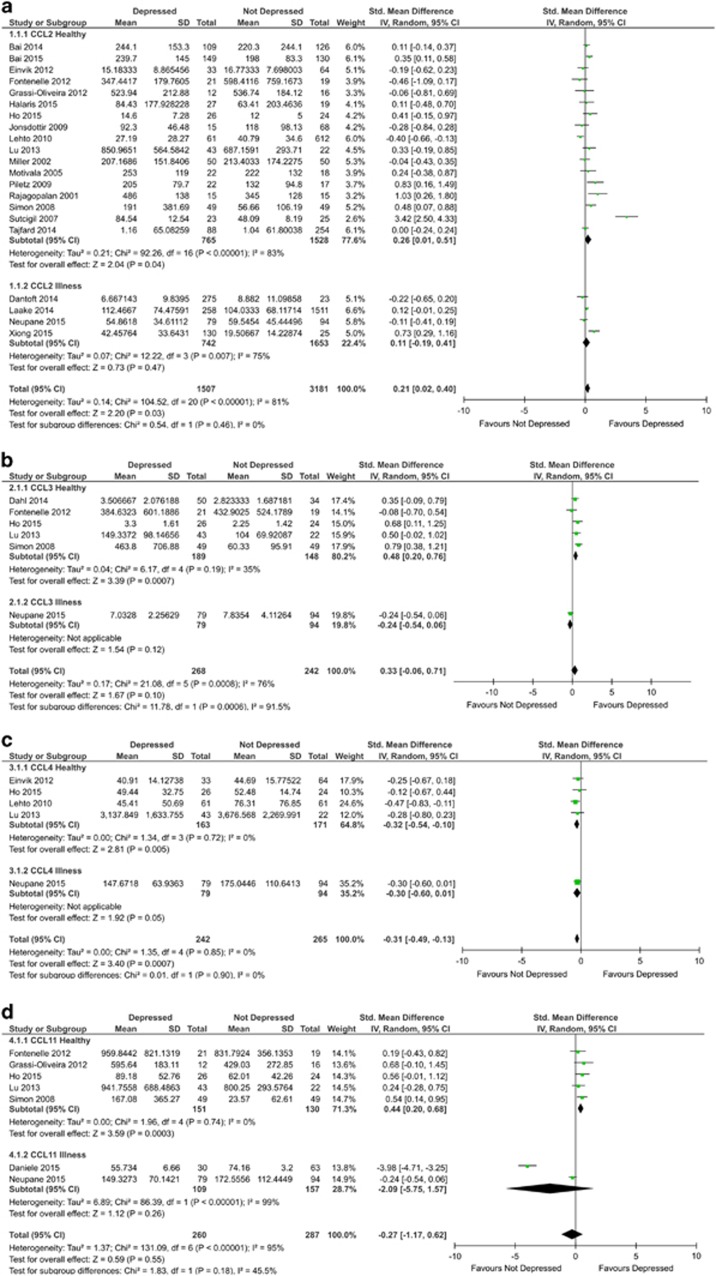
Forest plot of CCL2 (**a**), CCL3 (**b**), CCL4 (**c**) and CCL11 (**d**) chemokine levels in plasma and serum of depressed and not depressed patients.

A total of 21 studies including 4688 participants (1507 cases, 3181 control) were included in the CCL2 analysis. Blood CCL2 measurements were significantly higher in depressed subjects compared with controls (SMD=0.21; 95% CI: 0.02–0.40; *P*=0.03; Figure 2). Significant heterogeneity was observed in the included studies (*I*^2^=81% *P*<0.00001). Evidence of asymmetry was present in the funnel plot (Supplementary Figure 2) and Egger’s test demonstrated that this was significant (*P*=0.0071); despite this, trim and fill analysis did not impute any missing studies.

Sensitivity analyses demonstrated that in studies with physically healthy participants there was a significant difference in blood CCL2, similar to that observed across all studies (SMD=0.26; 95% CI: 0.01–0.51; *P*=0.04; Figure 2). Studies in the presence of illness did not reveal a significant difference. Comparisons of plasma and serum measurements revealed a significant difference in serum (SMD=0.33; 95% CI: 0.09–0.58; *P*=0.007), but not plasma. Analysis of studies with a low risk of bias did not retain statistical significance. Further details of analysis can be found in Supplementary Table 2.

#### CCL3

A total of 6 studies including 510 participants (268 cases, 242 controls) were included in the CCL3 analysis. There was no significant difference between blood CCL3 measurements in depressed and control subjects (SMD=0.33; 95% CI: −0.06 to 0.71; *P*=0.10; Figure 2). Significant heterogeneity was observed across the included studies (*I*^2^=76% *P*=0.0008). There was no obvious asymmetry in the funnel plot (Supplementary Figure 3) and Egger’s test was not significant (*P*=0.5472).

Sensitivity analyses demonstrated that in studies with physically healthy participants there was a significant increase in blood CCL3 between depressed and control subjects (SMD=0.48; 95% CI: 0.20–0.76; *P*=0.0007). Neither plasma nor serum retained significance alone. Analysis of studies with a low risk of bias (*n*=4) also demonstrated a significant difference between groups (SMD=0.58; 95% CI: 0.35–0.82; *P*<0.00001). Further details of analysis can be found in Supplementary Table 3.

#### CCL4

A total of 5 studies including 507 participants (242 cases, 265 controls) were included in the CCL4 analysis. Blood CCL4 measurements were significantly lower in depressed subjects compared with controls (SMD=−0.31; 95% CI: −0.49 to −0.13; *P*=0.0007; Figure 2). Evidence of heterogeneity across studies was not present (I^2^=0% *P*=0.85). There was no obvious asymmetry in the funnel plot (Supplementary Figure 4) and Egger’s test was not significant (*P*=0.5548).

Sensitivity analyses demonstrated that in studies with physically healthy participants there was a significant difference in blood CCL4, similar to that observed across all studies (SMD=−0.32; 95% CI: −0.54 to −0.10; *P*=0.005). Comparisons of plasma and serum measurements revealed a significant difference in serum (SMD=−0.33; 95% CI: −0.54 to −0.12; *P*=0.002), but not plasma. All healthy studies for CCL4 were considered to have low risk of bias, and therefore further sensitivity analysis was not performed. Further details of analysis can be found in Supplementary Table 4.

#### CCL11

A total of 7 studies including 454 participants (230 cases, 224 controls) were included in the CCL11 analysis. There was no significant difference between blood CCL11 measurements in depressed and control subjects (Figure 2). Moderate heterogeneity was observed in the included studies (*I*^2^=64% *P*=0.02). There was evidence of possible asymmetry in the funnel plot (Supplementary Figure 5). However, Egger’s test was not significant (*P*=0.4629).

Sensitivity analysis demonstrated that in studies with healthy participants there was a significant increase in blood CCL11 in depressed subjects compared with controls (SMD=0.44; 95% CI: 0.20–0.68; *P*=0.0003). Neither plasma nor serum was significant alone. Analysis of studies with a low risk of bias also demonstrated a significant difference between groups (SMD=0.48; 95% CI: 0.22–0.74; *P*=0.0003). Further details of analysis can be found in Supplementary Table 5.

#### CXCL4

**Figure 3:**
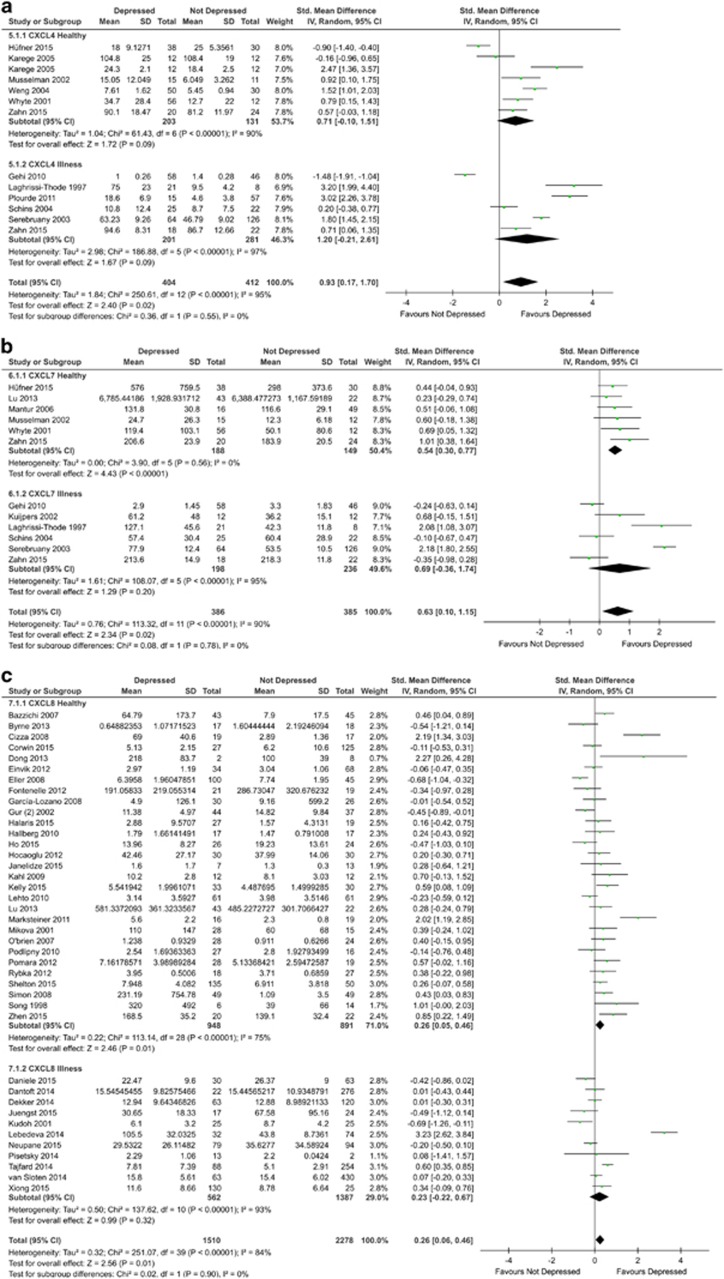
Forest plot of CXCL4 (**a**), CXCL7 (**b**) and CXCL8 (**c**) chemokine levels in plasma and serum of depressed and not depressed patients.

A total of 11 studies involving 792 participants were included in the blood CXCL4 meta-analysis. Blood CXCL4 measurements were significantly higher in depressed subjects compared with controls (SMD=1.03; 95% CI: 0.22–1.83; *P*=0.01; Figure 3). There was evidence of statistical heterogeneity across the studies (*I*^2^=96% *P*<0.00001). The funnel plot showed evidence of asymmetry (Supplementary Figure 6). However, the Egger’s test was not significant (*P*=0.3541).

Sensitivity analysis using Karege 2005 serum values instead of plasma values did not alter overall significance or statistical heterogeneity but reduced the overall SMD (SMD=0.82; 95% CI: 0.03–1.61; *P*=0.04). Sensitivity analysis involving only studies with physically healthy participants or only those with a physical illness did not attain statistical significance. Neither plasma nor serum was significant alone. Sensitivity analysis involving studies with a low risk of bias did not retain statistical significance. Further details of analysis can be found in Supplementary Table 6.

#### CXCL7

A total of 11 studies involving 771 participants were included in the blood CXCL7 meta-analysis. Blood CXCL7 measurements were significantly higher in depressed subjects compared with controls (SMD=0.63; 95% CI: 0.10–1.15; *P*=0.02; Figure 3). There was evidence of statistical heterogeneity across the studies (*I*^2^=90% *P*<0.00001). The funnel plot showed evidence of asymmetry (Supplementary Figure 7). However, the Egger’s test was not significant (*P*=0.7837).

Sensitivity analysis involving only studies with physically healthy participants demonstrated a significant increase in blood CXCL7 (SMD=0.54, 95% CI: 0.30–0.77; *P*<0.00001). However, this was not true for studies with only physically ill participants. Furthermore, among studies with only healthy participants there was no evidence of statistical heterogeneity (*I*^2^=0% *P*=0.56). Comparisons of plasma and serum measurements revealed a significant difference in plasma (SMD=0.71; 95% CI: 0.05–1.37; *P*=0.04), but not serum. Sensitivity analysis involving studies that demonstrated low risk of bias retained statistical significance for the healthy subgroup (SMD=0.52; 95% CI: 0.20–0.84; *P*=0.001) and borderline statistical significance overall (SMD=0.40; 95% CI: 0.00–0.80; *P*=0.05). Further details of analysis can be found in Supplementary Table 7.

#### CXCL8

A total of 40 studies involving 3788 participants were included in the blood CXCL8 meta-analysis. Blood CXCL8 measurements were significantly higher in depressed subjects compared with controls (SMD=0.26; 95% CI: 0.06–0.46; *P*=0.01; Figure 3). There was evidence of statistical heterogeneity across the studies (*I*^2^=84% *P*<0.00001). The funnel plot showed evidence of asymmetry (Supplementary Figure 8). However, the Egger’s test was not significant (*P*=0.1069). Removing the potential outlier among the studies with participants with physical illness, Lebedeva 2014,^73^ retained overall statistical significance (SMD=0.16; 95% CI: 0.00–0.32; *P*=0.04).

Sensitivity analysis involving only studies with physically healthy participants demonstrated a significant increase in blood CXCL8 (SMD=0.26; 95% CI: 0.05–0.46; *P*=0.01) but still with statistical heterogeneity (*I*^2^=75% *P*<0.00001). Comparisons of plasma and serum measurements revealed a significant difference in plasma (SMD=0.57; 95% CI: 0.18–0.96; *P*=0.004), but not serum. Given the large number of studies, it was possible to conduct sensitivity analysis for studies involving only female participants; this retained statistical significance overall (SMD=0.51; 95% CI: 0.03–1.00; *P*=0.04) and among healthy participants only (SMD=0.69; 95% CI: 0.06–1.33; *P*=0.03). Sensitivity analysis involving studies that demonstrated low risk of bias did not retain statistical significance. Further details of analysis can be found in Supplementary Table 8.

**Figure 4:**
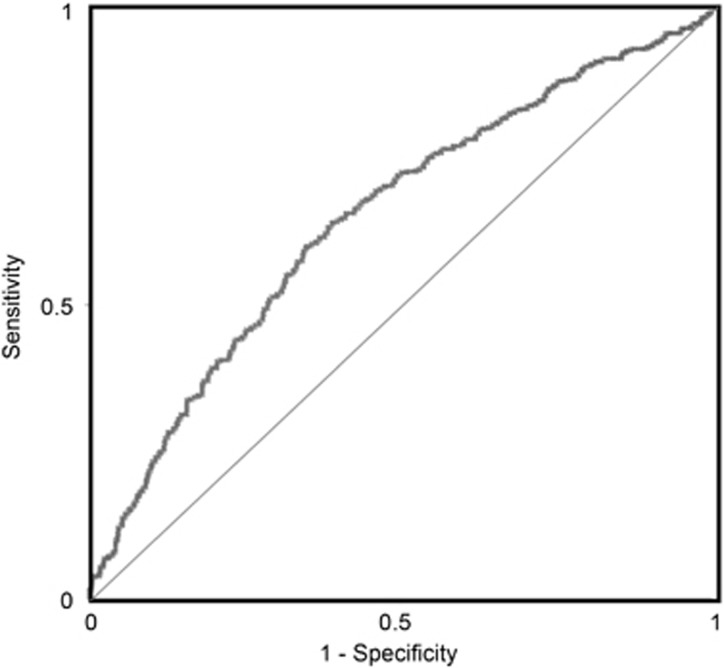
Receiver operating characteristic (ROC) curve based on effect size for plasma CXCL8 in studies with healthy participants gives an area under the curve of 64.5%.

Based on the effect size for plasma CXCL8 in studies with only physically healthy participants, CXCL8 produced a classification accuracy of 62.1% (95% CI: 58.9–65.14%), with receiver operating characteristic area under the curve of 64.5% (Figure 4); sensitivity of 63.4% (95% CI: 59.01–67.65%) and specificity of 60.63% (95% CI: 56.1–64.99%). Based on a population prevalence of 10% (MDD in the general population), the PPV was 15.18% and NPV was high at 93.71%.

### Additional sensitivity analyses

Additional sensitivity analyses of CCL3, CCL4, CCL11, CXCL4 and CXCL7 did not affect the significance of results. For CCL2, significance of results was not affected except when including only studies that matched for body mass index or for circadian rhythm of blood sampling. For CXCL8, all additional sensitivity analyses except removing Fontenelle 2012 resulted in a loss of overall significance. CXCL8 studies using DSM MDD diagnoses or those matching for age retained significance for healthy participants. When looking at only cardiovascular illness, CXCL8 meta-analysis attained statistical significance (SMD=0.80; 95% CI: 0.07–1.54; *P*=0.03). Detailed results of additional sensitivity are summarised in Supplementary Table 9.

### Other chemokines

We also examined blood CCL5, CCL7, CXCL9 and CXCL10, and CSF CXCL8 and CXCL10. However, no significant differences were observed in these analyses. Details of the studies and the calculated differences can be seen in Table 1.

## Discussion

Chemokine ligands and receptors are expressed throughout both the developing and mature central nervous system,^110, 111^ under both physiological and inflammatory conditions. This meta-analysis indicates that a number of these chemokines (CCL2, CCL3, CCL4, CCL11, CXCL4, CXCL7 and CXCL8) when measured in the blood discriminate between those with and without depression.

Specifically, significant increases were seen in CCL2, CXCL4, CXCL7 and CXCL8 and a decrease in CCL4 when comparing depressed and nondepressed. Examining our secondary outcomes, including only healthy participants showed additional increases in CCL3 and CCL11, whereas CXCL4 lost significance. There were no significant differences in chemokine levels when restricting our analysis to only those with physical comorbidities, implying that additional inflammatory change associated with depression is masked by the raised profile associated with the underlying physical disease. However, when looking at cardiovascular illness alone CXCL8 attained statistical significance. Perhaps, this finding is related to the reduction in heterogeneity with analysing only one illness subtype. Analysis of studies measuring in plasma versus serum did not reveal any particular pattern, suggesting there was no specific advantage in using plasma rather than serum in terms of differences detected. Finally, when examining studies with a low risk of bias we found that effects tended to be ameliorated or lose significance, suggesting that higher-risk studies are greater contributors to the effect sizes observed.

On comparing our findings with those observed in other meta-analyses by Eyre *et al.*^30^ and Köhler *et al.*,^112^ significant increases in CCL2 found in our study are similar. It is worth noting, however, that significance for CCL2 seen in Köhler *et al.*^112^ did not remain after exclusion of a study by Shen *et al.*^113^ We excluded this study before meta-analysis because of its high risk of bias and our inability to contact the authors. In contrast to either Eyre *et al.*^30^ or Köhler *et al.*,^112^ we did note increases in CXCL8. In addition, when including studies with only a low risk of bias, neither CCL2 nor CXCL8 were significantly different in our meta-analysis, suggesting that less methodologically rigorous studies may be contributing to the differences we identified.

Although Köhler *et al.*^112^ did not identify alterations in CCL3, our study indicated significant increases that were retained even when considering only those studies with a lower risk of bias. Another possible explanation for the differences in CCL2, CCL3 and CXCL8 findings between our study and the other meta-analyses is that we identified a greater number of studies for inclusion, possibly because of our more complete search strategy. Finally, it is worth highlighting that we were able to analyse an increased number of chemokines in this study, compared with previous meta-analyses. Although this may be because of our broad inclusion criteria, it highlights the need for comprehensive search strategies when examining chemokine literature and the strengths of using the multiple variant names of these molecules when searching databases.

Our analysis suggests that the classification accuracy, receiver operating characteristic area under the curve, sensitivity and specificity of plasma CXCL8 in discriminating depressed subjects from controls is significantly better than 50% (chance). Because of the low specificity of depressive mood symptoms, identification of a test with high NPV that can discriminate MDD from other conditions that have similar symptomatology could have potential clinical utility. With a realistic population prevalence of major depressive disorder of 10%, we found a high NPV of 93.71%. This univariate test compares favourably with a 97.59% NPV established via meta-analysis of major depression screening instruments in primary care.^114^ We propose that accuracy would be likely to improve with a multivariate approach, utilising a number of blood and mood measures. In addition, replicating our CXCL8 meta-analysis using the complementary methodological approach suggested by Fried and Kievit^36^ allows for a more accurate interpretation of our results and adds to the validity of our findings.

Several human studies point to relevant role for chemokines in the biology of depression. In the brains (dorsal anterior cingulate) of depressed suicides compared with sudden death matched controls, Torres-Platas *et al.*^115^ showed that monocytes traffic to the brain and congregate perivascularly in association with CCL2. Similarly, Pantazatos *et al.*^116^ used RNA-sequencing on post-mortem brain tissue (DLPFC-BA9) from MDD suicide (MDD-S) and compared with MDD non-suicide and controls. The greatest differences between MDD with and without suicide (that is, within the MDD group) were in molecular pathways that related to microglia and the immune system, in particular in genes for CCL2 and CCL4. The greatest difference between MDD and controls was seen in the chemokine CXCL8. Pantazatos *et al.*^116^ have highlighted the issue of chemokine pleiotropy and this is germane to the apparently counterintuitive finding of reduced levels of CCL4. In their post-mortem tissue samples, Pantazatos *et al.*^116^ also noted lower expression of CCL2 in the brains of MDD-S—in contrast with Torres-Platas *et al.*^115^ who found raised levels in MDD-S. However, the data in the literature varies here, with measurements in different tissue compartments and different patient groups contributing to sometimes opposing findings. Interestingly, studies that have noted lower CXCL8, CCL2 and CCL4 have all been in groups exhibiting suicidal behaviour.^63, 117, 118, 119^

### Limitations

There are a number of limitations to this systematic review and meta-analysis. We found that there was a reasonable risk of publication bias for a number of analyses, particularly when looking at funnel plots. Overall, a risk of reporting bias was present. However, by contacting authors directly for data we hope to have overcome the main issues associated with this. Strengths are that by including multiple databases and following PRISMA guidelines, it is unlikely that relevant studies were missed. In addition, two independent reviewers assessed the quality and bias of included studies.

In terms of individual studies, the quality was highly variable with notable differences in controlling for confounders, the mixture of plasma and serum used to measure the chemokines and the lack of parsing of subtypes of depression, for example, melancholic vs nonmelancholic.

### Future directions

If the relationships between chemokine biology and the neurobiology of depression are to be fully understood, then a number of steps require to be followed. Longitudinal studies of peripheral blood measures of chemokines would allow more than the currently available single time snapshots and help to unpick a state versus trait relationship with inflammatory biomarkers. Such designs would also allow correlation with age groups, symptom types and Research Domain Criteria profiles along with relationships, if any, with treatment status.

More fundamental mechanistic research is essential if we are to truly understand the role that chemokines play in the context of sickness behaviour and longer-term models of inflammation in preclinical models. Much of the work to date has focussed on the role that chemokines play in normal neurodevelopment. The extent to which chemokines play their part in orchestrating the systemic inflammatory response is strongly indicative of an important role in how this inflammatory response operates within the central nervous system thus affecting behaviour. Furthermore, unpicking inflammatory versus neuromodulatory functions and the relationship between them will provide further insight into the pathophysiological mechanisms of depressive disorders.

### Summary

In this systematic review and meta-analysis of the available literature, a number of chemokines were found to be altered in depressed individuals. These chemokines are involved in both neurobiological functions and with leukocyte trafficking and recruitment known to be associated with depression.

## Figures and Tables

**Table 1:** Summary table of effect estimates for blood chemokines with nonsignificant findings between depressed and nondepressed individuals

*Chemokine*	*Studies*	*Participants*	*Effect estimate (95% CI)*
*Blood (plasma/serum)*			
CCL5	7	251	−0.10 (−0.59, 0.40)
CCL7	3	156	0.07 (−0.65, 0.79)
CXCL9	3	161	−0.05 (−0.60, 0.49)
CXCL10	3	350	1.17 (−0.26, 2.61)
			
*CSF*			
CXCL8	6	361	0.19 (−0.15, 0.54)
CXCL10	4	208	−0.06 (−0.35, 0.22)

Abbreviations: CI, confidence interval; CSF, cerebrospinal fluid.
