# Supplementary material for: Chemokines in depression in health and in inflammatory illness: a systematic review and meta-analysis
Source: Mol Psychiatry. 2017 Nov 14;23(1):48–58. doi: 10.1038/mp.2017.205 (PMC5754468; doi:10.1038/mp.2017.205)
Supplement: Supplementary Table [file mp2017205x1.doc]

Search Strategy

| **Search** | **Query** |
| --- | --- |
| 1 | chemokine* |
| 2 | ccl1 or ccl2 or ccl3 or ccl4 or ccl5 or ccl6 or ccl7 or ccl8 or ccl9 or ccl10 or ccl11 or ccl12 or ccl13 or ccl14 or ccl15 or ccl16 or ccl17 or ccl18 or ccl19 or ccl20 or ccl21 or ccl22 or ccl23 or ccl24 or ccl25 or ccl26 or ccl27 or ccl28) |
| 3 | (cxcl1 or cxcl2 or cxcl3 or cxcl4 or cxcl5 or cxcl6 or cxcl7 or cxcl8 or cxcl9 or cxcl10 or cxcl11 or cxcl12 or cxcl13 or cxcl14 or cxcl15 or cxcl16 or cxcl17) |
| 4 | (xcl1 or xcl2) |
| 5 | cx3cl1 |
| 6 | (ccl or cxcl or xcl or cx3cl) |
| 7 | (scya1 or scya2 or scya3 or scya4 or scya5 or scya6 or scya7 or scya8 or scya9 or scya10 or scya11 or scya12 or scya13 or scya14 or scya15 or scya16 or scya17 or scya18 or scya19 or scya20 or scya21 or scya22 or scya23 or scya24 or scya25 or scya26 or scya27 or scya28) |
| 8 | (scyb1 or scyb2 or scyb3 or scyb4 or scyb5 or scyb6 or scyb7 or scyb8 or scyb9 or scyb10 or scyb11 or scyb12 or scyb13 or scyb14 or scyb15 or scyb16 or scyb17) |
| 9 | (scyc1 or scyc2) |
| 10 | sycd1 |
| 11 | (scya or scyb or scyc or scyd) |
| 12 | chemokine receptor* |
| 13 | (ccr1 or ccr2 or ccr2b or ccr3 or ccr4 or ccr5 or ccr6 or ccr7 or ccr8 or ccr9 or ccr10) |
| 14 | (cxcr1 or cxcr2 or cxcr3 or cxcr3b or cxcr4 or cxcr5 or cxcr6 or cxcr7) |
| 15 | xcr1 |
| 16 | cx3cr1 |
| 17 | (ccr or cxcr or xcr or cx3cr) |
| 18 | (chemotactic cytokine* or chemokine*) |
| 19 | (i-309 or i309 or tca-3 or tca3 or sise) |
| 20 | (monocyte chemotactic protein#1 or monocyte chemotactic protein-1 or mcp#1 or mcp-1 or small inducible cytokine a2 or gdcf#2 or gdcf-2 or hc11 or hsmcr30 or mcaf or cmc#cf or smc-cf) |
| 21 | (macrophage inflammatory protein#1a or macrophage inflammatory protein-1a or mip#1a or mip-1a or g0s19#1 or g0s19-1 or ld78alpha or mip#1#alpha or mip#1-alpha or mip-1#alpha or mip-1-alpha or mip1a) |
| 22 | (macrophage inflammatory protein#1# or macrophage inflammatory protein-1# or mip#1# or mip-1# or act2 or act-2 or at744#1 or g26 or g-26 or hc21 or lag-1 or lag1 or mip-1-beta or mip1b or mip1b1) |
| 23 | ((regulated on activation, normal t cell expressed and secreted) or rantes or d17s136e or sis-delta or sisd or tcp228 or eocp) |
| 24 | (c10 or mrp#1 or mrp-1 or mrp#2 or mrp-2) |
| 25 | (monocyte specific chemokine#3 or monocyte specific chemokine-3 or monocyte-specific chemokine#3 or monocyte-specific chemokine-3 or mcp#3 or fic or marc or mcp-3 or nc28) |
| 26 | (monocyte chemotactic protein#2 or monocyte chemotactic protein-2 or mcp-2 or mcp#2 or hc14) |
| 27 | (macrophage inflammatory protein#1#gamma or macrophage inflammatory protein-1#gamma or macrophage inflammatory protein#1-gamma or macrophage inflammatory protein-1-gamma or mip#1#gamma or mip#1-gamma or mip-1#gamma or mip-1-gamma or macrophage inflammatory protein#1* or macrophage inflammatory protein-1* or macrophage inflammatory protein-related protein-2 or mrp#2 or mrp-2 or ccf18) |
| 28 | (eosinophil chemotactic protein or eotaxin-1 or eotaxin#1) |
| 29 | (monocyte chemotactic protein 5 or mcp#5 or mcp-5 or mcp#1-related chemokine or mcp-1#related chemokine or mcp#1#related chemokine or mcp-1-related chemokine) |
| 30 | (mcp#4 or mcp-4 or ncc#1 or ncc-1 or ckbeta10 or scyl1 or ckb10) |
| 31 | (hcc#1 or hcc-1 or mcif or ckb1 or ckbeta1 or ncc#2 or ncc-2 or hcc#3 or hcc-3 or scyl2) |
| 32 | (leukotactin#1 or leukotactin-1 or mip#5 or mip-5 or hcc#2 or hcc-2 or ncc#3 or ncc-3 or scyl3 or lkn#1 or lkn-1 or mip#1d or mip-1d or hmrp#2b or hmrp-2b) |
| 33 | (lec or ncc#4 or ncc-4 or lmc or ckb12 or ckbeta12 or liver#expressed chemokine or liver-expressed chemokine or monotactin#1 or monotactin-1 or mtn#1 or mtn-1 or scyl4 or hcc#4 or hcc-4 or lcc#1 or lcc-1) |
| 34 | (tarc or dendrokine or abcd#2 or abcd-2 or (thymus and activation regulated chemokine) or a#152e5#3 or a-152e5#3) |
| 35 | (parc or dc-ck1 or dc#ck1 or amac-1 or amac#1 or ckb7 or ckbeta7 or mip#4 or mip-4 or (pulmonary and activation-regulated chemokine) or (pulmonary and activation#regulated chemokine) or dendritic cell-chemokine 1 or dendritic cell#chemokine 1 or alternative macrophage activation-associated cc chemokine-1 or alternative macrophage activation#associated cc chemokine-1 or alternative macrophage activation-associated cc chemokine#1 or alternative macrophage activation#associated cc chemokine#1 or macrophage inflammatory protein-4) |
| 36 | (elc or exodus#3 or exodus-3 or ckb11 or ckbeta11 or ebi1#ligand chemokine or ebi1-ligand chemokine or macrophage inflammatory protein-3-beta or macrophage inflammatory protein#3#beta or mip-3-beta or mip-3b or mip#3b) |
| 37 | (larc or exodus#1 or exodus-1 or ckb4 or ckbeta4 or liver activation regulated chemokine or macrophage inflammatory protein#3 or macrophage inflammatory protein-3 or mip#3#alpha or mip-3#alpha or mip#3-alpha or mip-3-alpha or mip-3a or mip#3a or st38) |
| 38 | (slc or 6ckine or exodus#2 or exodus-2 or ckb9 or ckbeta9 or tca-4 or tca#4 or lymphoid-tissue chemokine* or lymphoid#tissue chemokine*) |
| 39 | (mdc or dc#b-ck or dc#beta-ck or dc-b-ck or abcd#1 or abcd-1 or stcp#1 or stcp-1) |
| 40 | (mpif#1 or mpif-1 or ckb8 or ckbeta8 or mip#3 or mip-3 or macrophage inflammatory protein 3 or myeloid progenitor inhibitory factor 1) |
| 41 | (eotaxin#2 or eotaxin-2 or mpif#2 or mpif-2 or ckb6 or ckbeta6 or myeloid progenitor inhibitory factor 2 or eosinophil chemotactic protein 2) |
| 42 | (teck or ckb15 or ckbeta15 or thymus#expressed chemokine or thymus-expressed chemokine) |
| 43 | (eotaxin#3 or eotaxin-3 or mip#4a or mip-4a or imac or tsc#1 or tsc-1 or thymic stroma chemokine#1 or thymic stroma chemokine-1 or macrophage inflammatory protein 4#alpha or macrophage inflammatory protein 4-alpha or mip#4#alpha or mip#4-alpha or mip-4-alpha or mip-4#alpha) |
| 44 | (ctack or ilc or eskine or pesky or skinkine or eskine or il#11 r#alpha#locus chemokine or il-11 r-alpha-locus chemokine or cutaneous t#cell#attracting chemokine or cutaneous t-cell-attracting chemokine) |
| 45 | (mec or mucosae#associated epithelial chemokine mucosae-associated epithelial chemokine or cck1) |
| 46 | (gro#a or gro-a or gro#alpha or gro-alpha or gro1 or nap#3 or nap-3 or kc or neutrophil#activating protein 3 or neutrophil-activating protein 3 or melanoma growth stimulating activity alpha or msga#alpha or msga-alpha or msga#a or msga-a) |
| 47 | (gro#b or gro-b or gro#beta or gro-beta or gro#2 or mip#2a or mip-2a or macrophage inflammatory protein 2#alpha or macrophage inflammatory protein 2-alpha or mip2#alpha or mip2-alpha or growth#regulated protein beta or growth-regulated protein beta or gro oncogene#2 or gro oncogene-2 or gro-2 or mgsa#beta or mgsa-beta or mgsa#b or mgsa-b or cinc#2a or cinc-2a) |
| 48 | (gro#gamma or gro-gamma or gro#3 or gro-3 or mip#2b or mip-2b or mip#2beta or mip2-beta or gro3 oncogene or gro protein gamma or grog or macrophage inflammatory protein#2#beta or macrophage inflammatory protein#2-beta or macrophage inflammatory protein-2#beta or macrophage inflammatory protein-2-beta or cinc#2b or cinc-2b) |
| 49 | (pf-4 or platelet factor#4 or pf#4 or platelet factor-4) |
| 50 | (ena#78 or ena-78 or epithelial-derived neutrophil-activating peptide 78 or epithelial#derived neutrophil#activating peptide 78) |
| 51 | (gcp#2 or gcp-2 or granulocyte chemotactic protein#2 or granulocyte chemotactic protein-2 or cka#3 or cka-3) |
| 52 | (nap#2 or nap-2 or ctapiii or b-tg or beta-tg or b#tg or beta#tg or pep or beta-thromboglobulin or beta#thromboglobulin or pro#platelet basic protein or pro-platelet basic protein or ppbp or b#tg1 or b-tg1 or ctap#iii or ctap-iii or ctap#3 or ctap-3 or la#pf4 or la-pf4 or ldgf or mdgf or pbp or tc#1 or tc-1 or tc#2 or tc-2 or tgb or tgb#1 or tgb-1 or thbgb or thbgb#1 or thbgb-1) |
| 53 | (il-8 or nap-1 or mdncf or gcp-1 or gcp#1 or il#8 or interleukin 8 or neutrophil chemotactic factor or lect or luct or lynap or mdncf ormonap or naf or nap#1) |
| 54 | (mig or crg-10 or monokine induced by gamma interferon or cmk or humig or crg#10) |
| 55 | (ip-10 or ip#10 or crg#2 or crg-2 or interferon gamma-induced protein 10 or interferon gamma induced protein 10 or small-inducible cytokine b10 or small inducible cytokine b10 or c7 or ifi10 or inp10 or gip#10 or gip-10 or mob#1 or mob-1) |
| 56 | (i#tac or i-tac or h#174 or h-174 or ip#9 or ip-9 or scyb9b or b#r1 or b-r1 or beta#r1 or beta-r1 or interferon inducible t cell alpha chemoattractant or interferon-inducible t-cell alpha chemoattractant or interferon-gamma-inducible protein 9 or intergeron gamma inducible protein 9) |
| 57 | (sdf-1 or sdf#1 or tlsf or tpar1 or pbsf or stromal cell-derived factor 1 or stromal cell#derived factor 1 or irh) |
| 58 | (b lymphocyte chemoattractant or bca-1 or bca#1 or blr1l or blc or angie#) |
| 59 | ((breast and kidney-expressed chemokine) or brak or njac or bolekine or kec or mip-2g or mip#2g or bmac or ks1) |
| 60 | (lungkine or weche) |
| 61 | (sr#psox or sr-psox or cxclg16) |
| 62 | (dmc or vcc#1 or vcc-1 or vegf co#regulated chemokine 1 or vegf co-regulated chemokine 1 or (dendritic cell- and monocyte-attracting chemokine-like protein) or (dendritic cell and monocyte attracting chemokine-like protein) or (dendritic cell and monocyte attracting chemokine like protein)) |
| 63 | (lymphotactin or atac or ltn or lptn or scm#1# or scm-1#) |
| 64 | lymphotactin |
| 65 | (fractalkine or neurotactin or abcd#3 or abcd-3 or c3xkine or cxc3 or cxc3c or ntn or ntt) |
| 66 | (cluster of differentiation 191 or cd191 or ckr-1 or ckr#1 or cmkbr1 or hm145 or mip1ar or scyar1) |
| 67 | (ccr2# or cd192 or cluster of differentiation 192 or cc#ckr#2 or cc#ckr-2 or cc-ckr#2 or cc-ckr-2 or ccr#2 or ccr-2 or ckr-2# or ckr#2# or cmkbr2 or mcp#1#r or mcp-1#r or mcp#1-r or mcp-1-r) |
| 68 | (cd193 or cluster of differentiation 193 or cc#ckr#3 or cc-ckr#3 or cc#ckr-3 or cc-ckr-3 or ckr-3 or ckr#3 or cmkbr3) |
| 69 | (cd194 or cluster of differentiation 194 or cc#ckr#4 or cc#ckr-4 or cc-ckr#4 or cc-ckr-4 or ckr-4 or ckr#4 or cmkbr4 or chemr13 or hgcn:14099 or k5-5) |
| 70 | (cd195 or cluster of differentiation 195 or cc#ckr#5 or cc#ckr-5 or cc-ckr#5 or cc-ckr-5 or ccr#5 or ccr-5 or ckr-5 or ckr#5 or cmkbr5 or iddm22) |
| 71 | (cd196 or cluster of differentiation 196 or bn#1 or bn-1 or cc#ckr#6 or cc#ckr-6 or cc-ckr#6 or cc-ckr-6 or ccr#6 or ccr-6 or ckr-l3 or ckr#l3 or cmkbr6 or dcr2 or dry6 or g protein-coupled receptor 29 or g protein-coupled receptor cy4 or gpr29 or gprcy4 or strl22) |
| 72 | (cd197 or cluster of differentiation 197 or blr2 or cdw197 or cmkbr7 or ebi1) |
| 73 | (cdw198 or cluster of differentiation w198 or cc#ckr#8 or cc#ckr-8 or cc-ckr#8 or cc-ckr-8 or ccr#8 or ccr-8 or ckrl1 or cmkbr8 or cmkbrl2 or cy6 or g protein-coupled receptor cy6 or gprcy6 or ter1) |
| 74 | (cdw199 or cluster of differentiation w199 or cc#ckr#9 or cc#ckr-9 or cc-ckr#9 or cc-ckr-9 or g protein-coupled receptor 9-6 or gpr#9-6 or gpr-9-6 or g protein-coupled receptor 28 or gpr28) |
| 75 | (gpr2 or g protein-coupled receptor 2 or g protein coupled receptor 2) |
| 76 | (interleukin 8 receptor or il-8ra or cd181 or cluster of differentiation 181 or cc#ckr#1 or cc#ckr-1 or cc-ckr#1 or cc-ckr-1 or cd128 or cdw128a or ckr#1 or ckr-1 or cmkar1 or il8r1 or il8ra or il8rba) |
| 77 | (il8rb or cluster of differentiation 182 or cd182 or cdw128b or cmkar2 or il#8r2 or il-8r2 or il#8ra or il-8ra or il#8rb or il-8rb) |
| 78 | (g protein-coupled receptor 9 or cd183 or cluster of differentiation 182 or cxcr3#a or cxcr3-a or cxcr3#b or cxcr3-b or cd182 or cd183 or ckr#l2 or ckr-l2 or cmkar3 or gpr9 or ip10#r or ip10-r or mig-r or mig#r) |
| 79 | (fusin or cd184 or cluster of differentiation 184 or d2s201e or fb22 or hm89 or hsy3rr or lap-3 or lap#3 or lcr#1 or lcr-1 or lestr or npy3r or npyr# or npyy3r or whim) |
| 80 | (cd185 or cluster of differentiation 185 or burkitt lymphoma receptor 1 or blr#1 or blr-1 or mdr#15 or mdr-15) |
| 81 | (cd186 or cluster of differentiation 186 or bonzo or strl33 or tymstr) |
| 82 | (ackr#3 or ackr-3 or cmkor#1 or cmkor-1 or cxc-r7 or cxcr-7 or cxcr#7 or g protein-coupled receptor 159 or g protein coupled receptor 159 or gpr159 or rdc-1 or rdc#1) |
| 83 | (g protein-coupled receptor 5 or g protein coupled receptor 5 or gpr5 or cc#xcr#1 or cc-xcr#1 or cc#xcr-1 or cc-xcr-1) |
| 84 | (fractalkine receptor or g protein-coupled receptor 13 or g protein coupled receptor 13 or gpr13 or ccrl1 or cmkbrl1 or cmkdr1 or g protein-coupled receptor v28 or g protein coupled receptor v28 or gprv28 or v28) |
| 85 | S1 OR S2 OR S3 OR S4 OR S5 OR S6 OR S7 OR S8 OR S9 OR S10 OR S11 OR S12 OR S13 OR S14 OR S15 OR S16 OR S17 OR S18 OR S19 OR S20 OR S21 OR S22 OR S23 OR S24 OR S25 OR S26 OR S27 OR S28 OR S29 OR S30 OR S31 OR S32 OR S33 OR S34 OR S35 OR S36 OR S37 OR S38 OR S39 OR S40 OR S41 OR S42 OR S43 OR S44 OR S45 OR S46 OR S47 OR S48 OR S49 OR S50 OR S51 OR S52 OR S53 OR S54 OR S55 OR S56 OR S57 OR S58 OR S59 OR S60 OR S61 OR S62 OR S63 OR S64 OR S65 OR S66 OR S67 OR S68 OR S69 OR S70 OR S71 OR S72 OR S73 OR S74 OR S75 OR S76 OR S77 OR S78 OR S79 OR S80 OR S81 OR S82 OR S83 OR S84 |
| 86 | DE "Major Depression" OR DE "Anaclitic Depression" OR DE "Dysthymic Disorder" OR DE "Endogenous Depression" OR DE "Postpartum Depression" OR DE "Reactive Depression" OR DE "Recurrent Depression" OR DE "Treatment Resistant Depression" |
| 87 | depress* |
| 88 | (beck depression or bdi or bdi#1a or bdi-1a or bdi#ii or bdi-ii) |
| 89 | (burns depression or bdc) |
| 90 | (center for epidemiologic studies depression or ces-d or ces#d) |
| 91 | (cornell scale for depression in dementia or csdd) |
| 92 | (edinburgh post#natal depression or edinburgh post-natal depression or epds) |
| 93 | (geriatric depression or gds) |
| 94 | (hamilton rating scale for depression or hamilton depression rating scale or hrsd or hdrs or ham-d or ham#d) |
| 95 | (hospital anxiety and depression scale) |
| 96 | (inventory of depressive symptomatology or ids) |
| 97 | (inventory to diagnose depression or idd) |
| 98 | (kutcher adolescent depression or kads) |
| 99 | (major depress* or mdd or mdi) |
| 100 | (montgomery-asberg depression rating scale or montgomery asberg depression rating scale or madrs) |
| 101 | (patient health questionnaire-9 or phq-9 or patient health questionnaire#9 or phq#9) |
| 102 | (quick inventory of depressive symptomatology or qids) |
| 103 | (raskin depression rating scale or three-area severity of depression scale or three area severity of depression scale or rdrs) |
| 104 | (rads-2 or reynolds adolescent depression scale or rads#2 or rads or rcds-2 or rcds or rcds#2 or reynolds child depression scale) |
| 105 | (wechsler depression rating scale or wdrs) |
| 106 | (zung self-rating depression scale or zung self rating depression scale or sds or zsrds) |
| 107 | S86 OR S87 OR S88 OR S89 OR S90 OR S91 OR S92 OR S93 OR S94 OR S95 OR S96 OR S97 OR S98 OR S99 OR S100 OR S101 OR S102 OR S103 OR S104 OR S105 OR S106 |
| 108 | S85 AND S107 |
