# Supplementary material for: Chemokines in depression in health and in inflammatory illness: a systematic review and meta-analysis
Source: Mol Psychiatry. 2017 Nov 14;23(1):48–58. doi: 10.1038/mp.2017.205 (PMC5754468; doi:10.1038/mp.2017.205)
Supplement: Supplementary Table 1 [file mp2017205x2.doc]

| **Study ID** | **Study Design (Max. 3)** | **Outcomes  (Max. 6)** | **Confounders (Max. 6)** | **Other  (Max. 2)** | **Total Score** |
| --- | --- | --- | --- | --- | --- |
| Bai 2014 | 2 | 5 | 3 | 2 | 12 |
| Bai 2015 | 2 | 5 | 2 | 2 | 11 |
| Bazzichi 2007 | 2 | 5 | 3 | 2 | 12 |
| Blasko 2006 | 2 | 5 | 2 | 2 | 11 |
| Byrne 2013 | 3 | 5 | 3 | 2 | 13 |
| Cizza 2008 | 3 | 6 | 3 | 1 | 13 |
| Corwin 2015 | 3 | 5 | 6 | 1 | 15 |
| Dahl 2014 | 3 | 5 | 5 | 1 | 14 |
| Daniele 2015 | 3 | 5 | 1 | 1 | 10 |
| Dantoft 2014 | 2 | 5 | 1 | 2 | 10 |
| Dekker 2014 | 2 | 5 | 3 | 1 | 11 |
| Dong 2013 | 2 | 5 | 1 | 1 | 9 |
| Einvik 2012 | 3 | 5 | 4 | 1 | 13 |
| Eller 2008 | 2 | 5 | 5 | 2 | 14 |
| Fontenelle 2012 | 3 | 4 | 1 | 2 | 10 |
| García-Lozano 2008 | 3 | 5 | 1 | 0 | 9 |
| Gehi 2010 | 3 | 5 | 2 | 2 | 12 |
| Grassi-Oliveira 2012 | 2 | 5 | 3 | 2 | 12 |
| Gur 2002 | 3 | 5 | 1 | 2 | 11 |
| Halaris 2015 | 3 | 4 | 5 | 1 | 13 |
| Hallberg 2010 | 3 | 5 | 3 | 2 | 13 |
| Ho 2015 | 2 | 5 | 4 | 2 | 13 |
| Hocaoglu 2012 | 3 | 4 | 3 | 2 | 12 |
| Hüfner 2015 | 3 | 5 | 5 | 2 | 15 |
| Janelidze 2013 | 3 | 5 | 1 | 1 | 10 |
| Janelidze 2015 | 3 | 3 | 5 | 2 | 13 |
| Jonsdottir 2009 | 3 | 3 | 3 | 2 | 11 |
| Juengst 2015 | 2 | 5 | 4 | 2 | 13 |
| Kahl 2009 | 2 | 5 | 6 | 2 | 15 |
| Karege 2005 | 3 | 5 | 2 | 2 | 12 |
| Kelly 2015 | 2 | 5 | 2 | 1 | 10 |
| Kern 2014 | 3 | 5 | 2 | 2 | 12 |
| Kudoh 2001 | 3 | 1 | 1 | 2 | 7 |
| Kuijpers 2002 | 3 | 5 | 3 | 2 | 13 |
| Laake 2014 | 3 | 5 | 1 | 1 | 10 |
| Laghrissi-Thode 1997 | 3 | 4 | 3 | 2 | 12 |
| Lebedeva 2014 | 2 | 1 | 0 | 0 | 3 |
| Lee 2009 | 3 | 4 | 4 | 2 | 13 |
| Lehto 2010 | 3 | 4 | 4 | 2 | 13 |
| Lindqvist 2009 | 3 | 5 | 4 | 2 | 14 |
| Lindqvist 2011 | 3 | 5 | 3 | 2 | 13 |
| Lu 2013 | 3 | 5 | 5 | 2 | 15 |
| Mantur 2006 | 3 | 5 | 1 | 2 | 11 |
| Marksteiner 2011 | 1 | 5 | 1 | 2 | 9 |
| Mikova 2001 | 2 | 5 | 4 | 2 | 13 |
| Miller 2002 | 3 | 3 | 4 | 2 | 12 |
| Motivala 2005 | 3 | 5 | 6 | 2 | 16 |
| Musselman 2002 | 3 | 6 | 2 | 2 | 13 |
| Neupane 2015 | 3 | 5 | 0 | 1 | 9 |
| O'brien 2007 | 3 | 5 | 2 | 2 | 12 |
| Oglodek 2014 | 2 | 2 | 4 | 1 | 9 |
| Piletz 2009 | 3 | 6 | 6 | 2 | 17 |
| Pisetsky 2014 | 2 | 3 | 1 | 2 | 8 |
| Plourde 2011 | 2 | 5 | 0 | 1 | 8 |
| Podlipny 2010 | 3 | 4 | 3 | 1 | 11 |
| Pomara 2012 | 2 | 5 | 0 | 1 | 8 |
| Pomara 2013 | 2 | 5 | 0 | 1 | 8 |
| Rajagopalan 2001 | 2 | 6 | 4 | 2 | 14 |
| Rybka 2012 | 0 | 4 | 2 | 1 | 7 |
| Schins 2004 | 3 | 5 | 4 | 2 | 14 |
| Serebruany 2003 | 3 | 4 | 1 | 2 | 10 |
| Shelton 2015 | 3 | 5 | 1 | 2 | 11 |
| Simon 2008 | 2 | 5 | 3 | 2 | 12 |
| Song 1998 | 2 | 5 | 3 | 2 | 12 |
| Sutcigil 2007 | 2 | 2 | 3 | 2 | 9 |
| Tajfard 2014 | 3 | 5 | 2 | 1 | 11 |
| van Sloten 2014 | 3 | 5 | 2 | 1 | 11 |
| Weng 2004 | 2 | 5 | 0 | 1 | 8 |
| Whyte 2001 | 3 | 4 | 2 | 2 | 11 |
| Wong 2008 | 3 | 1 | 4 | 2 | 10 |
| Xiong 2015 | 3 | 6 | 1 | 1 | 11 |
| Zahn 2015 | 3 | 5 | 3 | 2 | 13 |
| Zhen 2015 | 3 | 5 | 1 | 2 | 11 |

Supplementary Table 1. Quality Scores
