# Supplementary material for: Chemokines in depression in health and in inflammatory illness: a systematic review and meta-analysis
Source: Mol Psychiatry. 2017 Nov 14;23(1):48–58. doi: 10.1038/mp.2017.205 (PMC5754468; doi:10.1038/mp.2017.205)
Supplement: Supplementary Table 2 [file mp2017205x3.doc]

| **Outcome or Subgroup** | **Studies** | **Participants** | **Effect Estimate [95% C.I]** |
| --- | --- | --- | --- |
| 1.1 CCL2 Plasma/Serum | 21 | 4688 | 0.21 [0.02, 0.40] |
| 1.1.1 CCL2 Healthy | 17 | 2293 | 0.26 [0.01, 0.51] |
| 1.1.2 CCL2 Illness | 4 | 2395 | 0.11 [-0.19, 0.41] |
| 1.2 CCL2 Plasma | 8 | 696 | -0.22 [-0.74, 0.30] |
| 1.2.1 CCL2 Plasma Healthy | 7 | 398 | -0.22 [-0.85, 0.41] |
| 1.2.2 CCL2 Plasma Illness | 1 | 298 | -0.22 [-0.65, 0.20] |
| 1.3 CCL2 Serum | 13 | 3992 | 0.33 [0.09, 0.58] |
| 1.3.1 CCL2 Serum Healthy | 10 | 1895 | 0.42 [0.07, 0.77] |
| 1.3.2 CCL2 Serum Illness | 3 | 2097 | 0.20 [-0.15, 0.55] |

Supplementary Table 2. Sensitivity analyses of CCL2 Levels in plasma and serum samples of depressed and not depressed subjects.
