# Supplementary material for: Chemokines in depression in health and in inflammatory illness: a systematic review and meta-analysis
Source: Mol Psychiatry. 2017 Nov 14;23(1):48–58. doi: 10.1038/mp.2017.205 (PMC5754468; doi:10.1038/mp.2017.205)
Supplement: Supplementary Table 3 [file mp2017205x4.doc]

| **Outcome or Subgroup** | **Studies** | **Participants** | **Effect Estimate [95% C.I]** |
| --- | --- | --- | --- |
| 2.1 CCL3 Plasma/Serum | 6 | 510 | 0.33 [-0.06, 0.71] |
| 2.1.1 CCL3 Healthy | 5 | 337 | 0.48 [0.20, 0.76] |
| 2.1.2 CCL3 Illness | 1 | 173 | -0.24 [-0.54, 0.06] |
| 2.2 CCL3 Plasma | 3 | 189 | 0.30 [-0.00, 0.60] |
| 2.2.1 CCL3 Plasma Healthy | 3 | 189 | 0.30 [-0.00, 0.60] |
| 2.3 CCL3 Serum | 3 | 321 | 0.39 [-0.34, 1.13] |
| 2.3.1 CCL3 Serum Healthy | 2 | 148 | 0.75 [0.42, 1.09] |
| 2.3.2 CCL3 Serum Illness | 1 | 173 | -0.24 [-0.54, 0.06] |
| 2.4 CCL3 Low Bias | 4 | 297 | 0.58 [0.35, 0.82] |
| 2.4.1 CCL3 Low Bias Healthy | 4 | 297 | 0.58 [0.35, 0.82] |

Supplementary Table 3. Sensitivity analyses of CCL3 Levels in plasma and serum samples of depressed and not depressed subjects.
