# Supplementary material for: Chemokines in depression in health and in inflammatory illness: a systematic review and meta-analysis
Source: Mol Psychiatry. 2017 Nov 14;23(1):48–58. doi: 10.1038/mp.2017.205 (PMC5754468; doi:10.1038/mp.2017.205)
Supplement: Supplementary Table 4 [file mp2017205x5.doc]

| **Outcome or Subgroup** | **Studies** | **Participants** | **Effect Estimate [95% C.I]** |
| --- | --- | --- | --- |
| 3.1 CCL4 Plasma/Serum | 5 | 507 | -0.31 [-0.49, -0.13] |
| 3.1.1 CCL4 Healthy | 4 | 334 | -0.32 [-0.54, -0.10] |
| 3.1.2 CCL4 Illness | 1 | 173 | -0.30 [-0.60, 0.01] |
| 3.2 CCL4 Plasma | 2 | 162 | -0.26 [-0.59, 0.07] |
| 3.2.1 CCL4 Plasma Healthy | 2 | 162 | -0.26 [-0.59, 0.07] |
| 3.3 CCL4 Serum | 3 | 345 | -0.33 [-0.54, -0.12] |
| 3.3.1 CCL4 Serum Healthy | 2 | 172 | -0.36 [-0.68, -0.04] |
| 3.3.2 CCL4 Serum Illness | 1 | 173 | -0.30 [-0.60, 0.01] |

Supplementary Table 4. Sensitivity analyses of CCL4 Levels in plasma and serum samples of depressed and not depressed subjects.
