# Supplementary material for: Chemokines in depression in health and in inflammatory illness: a systematic review and meta-analysis
Source: Mol Psychiatry. 2017 Nov 14;23(1):48–58. doi: 10.1038/mp.2017.205 (PMC5754468; doi:10.1038/mp.2017.205)
Supplement: Supplementary Table 5 [file mp2017205x6.doc]

| **Outcome or Subgroup** | **Studies** | **Participants** | **Effect Estimate [95% C.I]** |
| --- | --- | --- | --- |
| 5.1 CCL11 Plasma/Serum | 7 | 547 | -0.27 [-1.17, 0.62] |
| 5.1.1 CCL11 Healthy | 5 | 281 | 0.44 [0.20, 0.68] |
| 5.1.2 CCL11 Illness | 2 | 266 | -2.09 [-5.75, 1.57] |
| 5.2 CCL11 Plasma | 3 | 133 | 0.32 [-0.04, 0.67] |
| 5.2.1 CCL11 Plasma Healthy | 3 | 133 | 0.32 [-0.04, 0.67] |
| 5.3 CCL11 Serum | 4 | 414 | -0.75 [-2.22, 0.72] |
| 5.3.1 CCL11 Serum Healthy | 2 | 148 | 0.55 [0.22, 0.88] |
| 5.3.2 CCL11 Serum Illness | 2 | 266 | -2.09 [-5.75, 1.57] |
| 5.4 CCL11 Low Bias | 4 | 241 | 0.48 [0.22, 0.74] |
| 5.4.1 CCL11 Low Bias Healthy | 4 | 241 | 0.48 [0.22, 0.74] |

Supplementary Table 5. Sensitivity analyses of CCL11 Levels in plasma and serum samples of depressed and not depressed subjects.
