# Supplementary material for: Chemokines in depression in health and in inflammatory illness: a systematic review and meta-analysis
Source: Mol Psychiatry. 2017 Nov 14;23(1):48–58. doi: 10.1038/mp.2017.205 (PMC5754468; doi:10.1038/mp.2017.205)
Supplement: Supplementary Table 6 [file mp2017205x7.doc]

| **Outcome or Subgroup** | **Studies** | **Participants** | **Effect Estimate [95% C.I]** |
| --- | --- | --- | --- |
| 6.1 CXCL4 Plasma/Serum | 11 | 792 | 1.03 [0.22, 1.83] |
| 6.1.1 CXCL4 Healthy | 6 | 310 | 0.85 [-0.05, 1.75] |
| 6.1.2 CXCL4 Illness | 6 | 482 | 1.20 [-0.21, 1.83] |
| 6.2 CXCL4 Plasma | 9 | 636 | 0.90 [-0.08, 1.88] |
| 6.2.1 CXCL4 Plasma Healthy | 5 | 266 | 0.92 [-0.21, 2.04] |
| 6.2.2 CXCL4 Plasma Illness | 4 | 370 | 0.88 [-0.98, 2.75] |
| 6.3 CXCL4 Serum | 2 | 108 | 0.43 [-0.05, 0.91] |
| 6.3.1 CXCL4 Serum Healthy | 2 | 68 | 0.26 [-0.45, 0.97] |
| 6.3.2 CXCL4 Serum Illness | 1 | 40 | 0.71 [0.06, 1.35] |
| 6.4 CXCL4 Low Bias | 7 | 382 | 0.63 [-0.30, 1.56] |
| 6.4.1 CXCL4 Low Bias Healthy | 4 | 162 | 0.70 [-0.57, 1.98] |
| 6.4.2 CXCL4 Low Bias Illness | 4 | 220 | 0.58 [-0.98, 2.14] |

Supplementary Table 6. Sensitivity analyses of CXCL4 Levels in plasma and serum samples of depressed and not depressed subjects.
