# Supplementary material for: Chemokines in depression in health and in inflammatory illness: a systematic review and meta-analysis
Source: Mol Psychiatry. 2017 Nov 14;23(1):48–58. doi: 10.1038/mp.2017.205 (PMC5754468; doi:10.1038/mp.2017.205)
Supplement: Supplementary Table 7 [file mp2017205x8.doc]

| **Outcome or Subgroup** | **Studies** | **Participants** | **Effect Estimate [95% C.I]** |
| --- | --- | --- | --- |
| 7.1 CXCL7 Plasma/Serum | 11 | 771 | 0.63 [0.10, 1.15] |
| 7.1.1 CXCL7 Healthy | 6 | 337 | 0.54 [0.30, 0.77] |
| 7.1.2 CXCL7 Illness | 6 | 434 | 0.69 [-0.36, 1.74] |
| 7.2 CXCL7 Plasma | 9 | 622 | 0.71 [0.05, 1.37] |
| 7.2.1 CXCL7 Plasma Healthy | 4 | 228 | 0.45 [0.16, 0.73] |
| 7.2.2 CXCL7 Plasma Illness | 5 | 394 | 0.90 [-0.29, 2.09] |
| 7.3 CXCL7 Serum | 2 | 149 | 0.39 [-0.36, 1.14] |
| 7.3.1 CXCL7 Serum Healthy | 2 | 109 | 0.74 [0.25, 1.23] |
| 7.3.2 CXCL7 Serum Illness | 1 | 40 | -0.35 [-0.98, 0.28] |
| 7.4 CXCL7 Low Bias | 8 | 448 | 0.40 [-0.00, 0.80] |
| 7.4.1 CXCL7 Low Bias Healthy | 4 | 204 | 0.52 [0.20, 0.84] |
| 7.4.2 CXCL7 Low Bias Illness | 5 | 244 | 0.31 [-0.35, 0.97] |

Supplementary Table 7. Sensitivity analyses of CXCL7 Levels in plasma and serum samples of depressed and not depressed subjects.
