# Supplementary material for: Chemokines in depression in health and in inflammatory illness: a systematic review and meta-analysis
Source: Mol Psychiatry. 2017 Nov 14;23(1):48–58. doi: 10.1038/mp.2017.205 (PMC5754468; doi:10.1038/mp.2017.205)
Supplement: Supplementary Table 8 [file mp2017205x9.doc]

| **Outcome or Subgroup** | **Studies** | **Participants** | **Effect Estimate [95% C.I]** |
| --- | --- | --- | --- |
| 8.1 CXCL8 Plasma/Serum | 40 | 3788 | 0.26 [0.06, 0.46] |
| 8.1.1 CXCL8 Healthy | 29 | 1839 | 0.26 [0.05, 0.46] |
| 8.1.2 CXCL8 Illness | 11 | 1949 | 0.23 [-0.22, 0.67] |
| 8.2 CXCL8 Plasma | 18 | 1429 | 0.57 [0.18, 0.96] |
| 8.2.1 CXCL8 Plasma Healthy | 15 | 975 | 0.46 [0.17, 0.75] |
| 8.2.2 CXCL8 Plasma Illness | 3 | 454 | 0.85 [-1.32, 3.01] |
| 8.3 CXCL8 Serum | 20 | 2302 | -0.01 [-0.20, 0.19] |
| 8.3.1 CXCL8 Serum Healthy | 13 | 822 | -0.02 [-0.28, 0.24] |
| 8.3.2 CXCL8 Serum Illness | 7 | 1480 | 0.02 [-0.27, 0.31] |
| 8.4 CXCL8 Low Bias | 18 | 1213 | 0.15 [-0.10, 0.41] |
| 8.4.1 CXCL8 Low Bias Healthy | 17 | 1172 | 0.19 [-0.07, 0.45] |
| 8.4.2 CXCL8 Low Bias Illness | 1 | 41 | -0.49 [-1.12, 0.14] |
| 8.5 CXCL8 Females | 8 | 679 | 0.51 [0.03, 1.00] |
| 8.5.1 CXCL8 Females Healthy | 6 | 266 | 0.69 [0.06, 1.33] |
| 8.5.2 CXCL8 Females Illness | 2 | 313 | 0.01 [-0.40, 0.43] |

Supplementary Table 8. Sensitivity analyses of CXCL8 Levels in plasma and serum samples of depressed and not depressed subjects.
