# Supplementary material for: Chemokines in depression in health and in inflammatory illness: a systematic review and meta-analysis
Source: Mol Psychiatry. 2017 Nov 14;23(1):48–58. doi: 10.1038/mp.2017.205 (PMC5754468; doi:10.1038/mp.2017.205)
Supplement: Supplementary Table 9 [file mp2017205x10.doc]

**Further sensitivity analyses**

| **Analysis** | **Studies (n)** | **Depressed (n)** | **Not Depressed (n)** | **Effect Size** | **C.I.** | **p Value** |
| --- | --- | --- | --- | --- | --- | --- |
| **CCL2** | | | | | | |
| DSM Diagnosis Only | 16 | 753 | 1349 | 0.30 | 0.03, 0.56 | 0.03 |
| Sub-types of illness | Not performed as not enough studies | | | | | |
| Age | 15 | 656 | 1255 | 0.33 | 0.05, 0.62 | 0.02 |
| Gender | 16 | 931 | 1278 | 0.29 | 0.02, 0.57 | 0.03 |
| BMI | 10 | 375 | 962 | 0.19 | -0.08, 0.47 | 0.17 |
| Smoking | 6 | 166 | 161 | 0.36 | 0.00, 0.71 | 0.05 |
| Circadian | 11 | 651 | 2610 | 0.04 | -0.14, 0.22 | 0.70 |
| Medication | 5 | 166 | 159 | 0.91 | 0.06, 1.76 | 0.04 |
| Articles  Only | 20 | 1428 | 3087 | 0.23 | 0.04, 0.43 | 0.02 |
| Remove Fontenelle | 20 | 1486 | 3162 | 0.24 | 0.05, 0.43 | 0.01 |
| Remove  Data Extraction | 16 | 971 | 1308 | 0.26 | -0.00, 0.53 | 0.05 |
| **CCL3** | | | | | | |
| DSM Diagnosis Only | All used DSM-IV criteria | | | | | |
| Sub-types of Illness | Not performed as not enough studies | | | | | |
| Articles  Only | Same as only healthy studies. (Neupane is the only illness study and is also only abstract) | | | | | |
| Remove  Fontenelle | 5 | 247 | 223 | 0.39 | -0.04, 0.83 | 0.07 |
| Remove Data Extraction | 5 | 218 | 208 | 0.32 | -0.15, 0.79 | 0.18 |
| **CCL4** | | | | | | |
| DSM Diagnosis Only | All used DSM-IV Criteria | | | | | |
| Sub-types of Illness | Not performed as not enough studies | | | | | |
| Article  Only | Same as only healthy studies. (Neupane is the only illness study and is also only abstract) | | | | | |
| Remove Data Extraction | 4 | 209 | 201 | -0.32, | -0.52,  -0.13 | 0.001 |
| **CCL11** | | | | | | |
| DSM Diagnosis Only | 6 | 230 | 224 | 0.28 | -0.06, 0.62 | 0.10 |
| Sub-types of Illness | Not performed as not enough studies | | | | | |
| Article  Only | Same as only healthy studies. (Neupane & Daniele both illness and were the only abstracts.) | | | | | |
| Remove  Fontenelle | 6 | 239 | 268 | -0.35 | -1.38, 0.68 | 0.51 |
| Remove  Data Extraction | None of the studies had data extraction | | | | | |
| **CXCL4** | | | | | | |
| DSM Diagnosis Only | 10 | 354 | 382 | 0.89 | 0.06, 1.71 | 0.04 |
| Sub-types of Illness:  Cardiovascular Disease only | 5 | 183 | 259 | 1.31 | -0.39, 3.01 | 0.13 |
| Article  Only | All articles | | | | | |
| Remove Data Extraction | None of the studies had data extraction | | | | | |
| **CXCL7** | | | | | | |
| DSM Diagnosis Only | 10 | 370 | 336 | 0.64 | 0.06, 1.21 | 0.03 |
| Sub-types of Illness:  Cardiovascular Disease only | 5 | 180 | 214 | 0.90 | -0.29, 2.09 | 0.14 |
| Article  Only | All articles | | | | | |
| Remove Data Extraction | None of the studies had data extraction | | | | | |
| **CXCL8** | | | | | | |
| DSM Diagnosis Only | 21 | 823 | 693 | 0.17 | -0.05, 0.39 | 0.13 |
| Sub-types of illness:  Cardiovascular Disease only | 5 | 376 | 903 | 0.80 | 0.07, 1.54 | 0.03 |
| Age | 22 | 711 | 1136 | 0.20 | -0.04, 0.44 | 0.10 |
| Gender | 22 | 684 | 982 | 0.11 | -0.11, 0.33 | 0.34 |
| BMI | 9 | 478 | 769 | 0.04 | -0.21, 0.29 | 0.74 |
| Smoking | 9 | 206 | 280 | 0.27 | -0.15, 0.70 | 0.21 |
| Circadian | 22 | 742 | 1007 | 0.08 | -0.11, 0.28 | 0.41 |
| Medication | 5 | 216 | 246 | 0.09 | -0.42, 0.61 | 0.72 |
| Article  Only | 32 | 1240 | 1923 | 0.15 | -0.03, 0.33 | 0.10 |
| Remove Fontenelle | 39 | 1489 | 2259 | 0.27 | 0.07, 0.47 | 0.008 |
| Remove Data Extraction | 28 | 979 | 1202 | 0.05 | -0.12, 0.22 | 0.55 |
