# Supplementary figures and images for: Chemokines in depression in health and in inflammatory illness: a systematic review and meta-analysis
Source: Mol Psychiatry. 2017 Nov 14;23(1):48–58. doi: 10.1038/mp.2017.205 (PMC5754468; doi:10.1038/mp.2017.205)

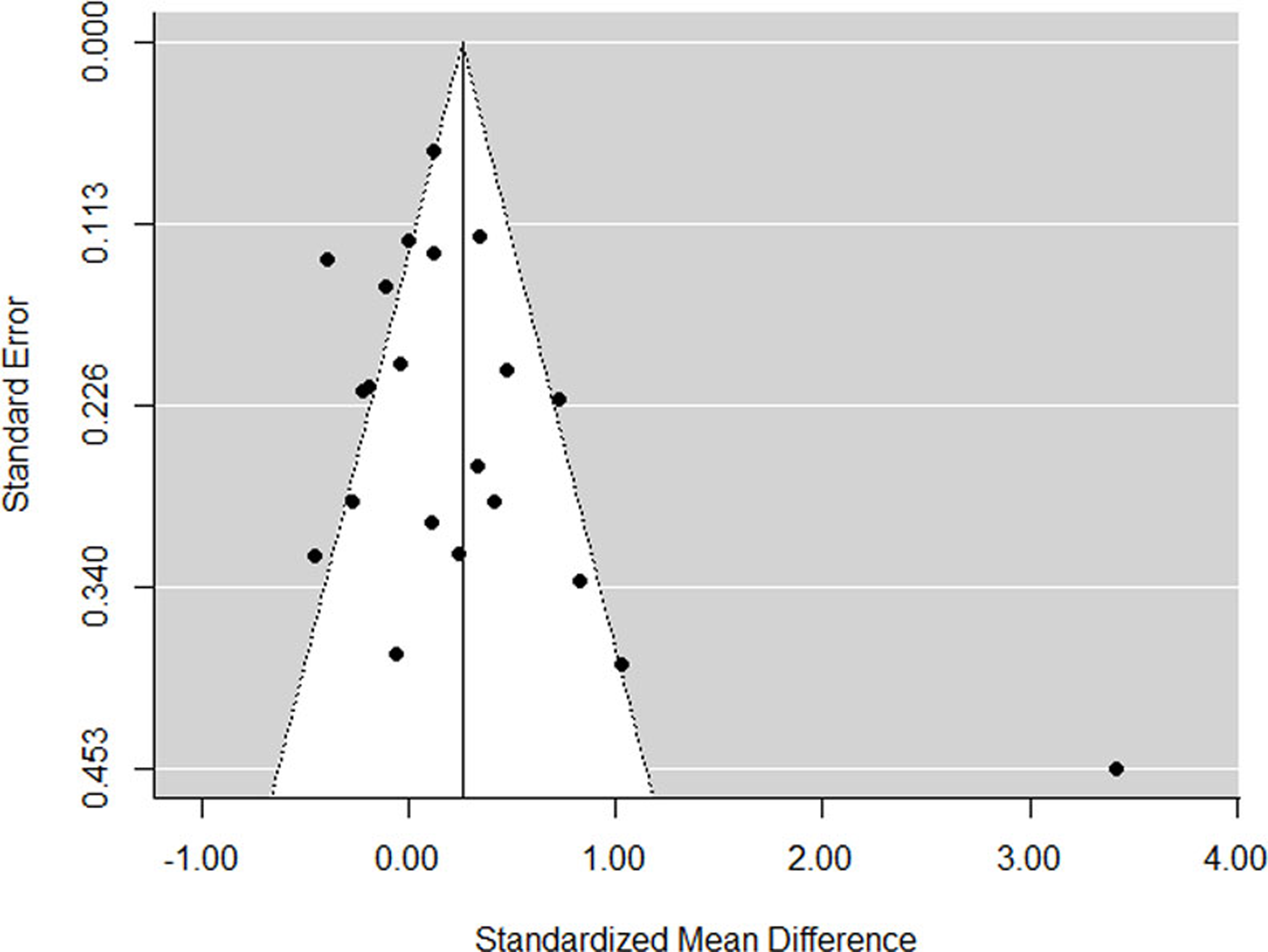

Supplement: Supplementary Figure 1 [file mp2017205x11.tif]

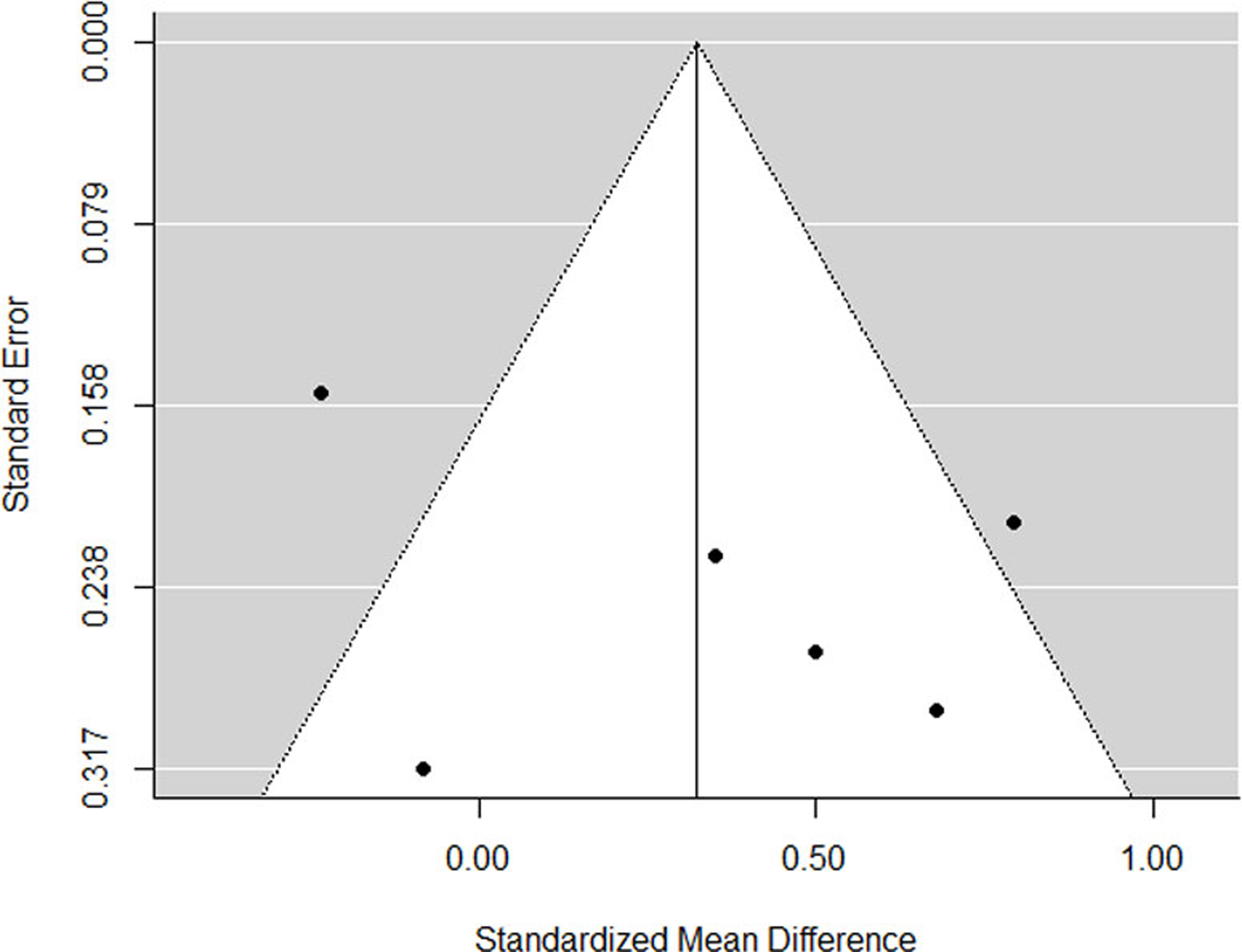

Supplement: Supplementary Figure 2 [file mp2017205x12.tif]

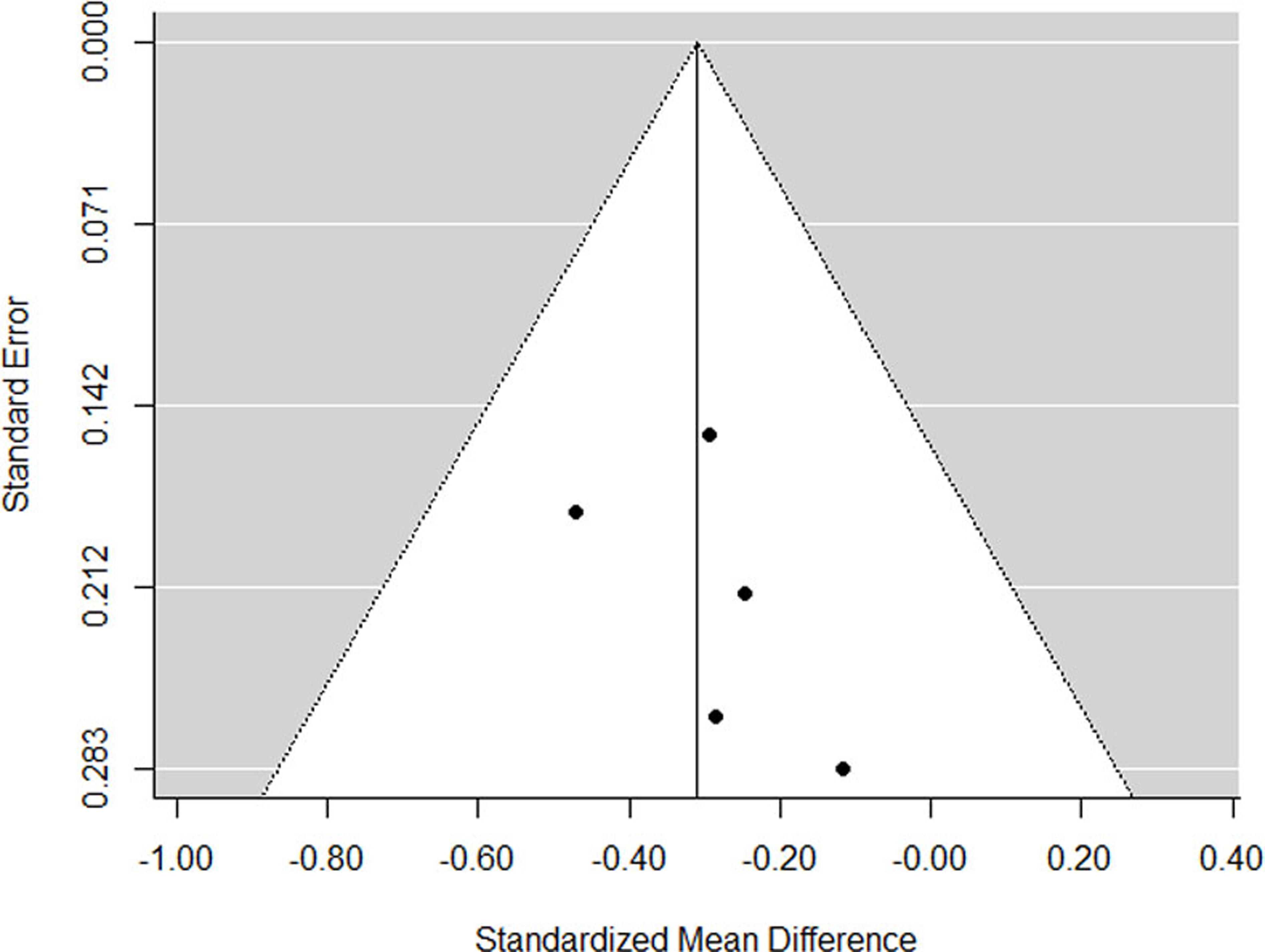

Supplement: Supplementary Figure 3 [file mp2017205x13.tif]

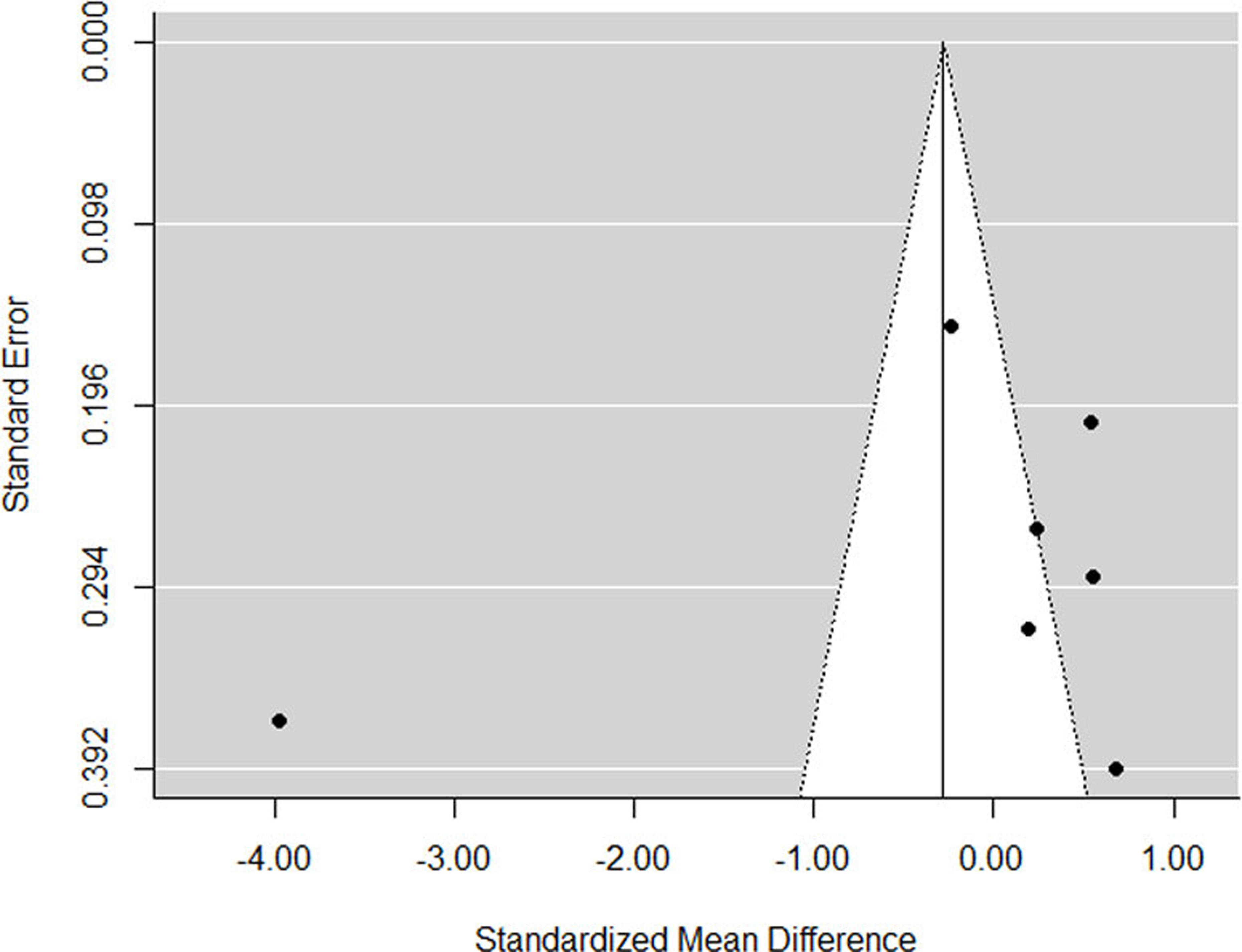

Supplement: Supplementary Figure 4 [file mp2017205x14.tif]

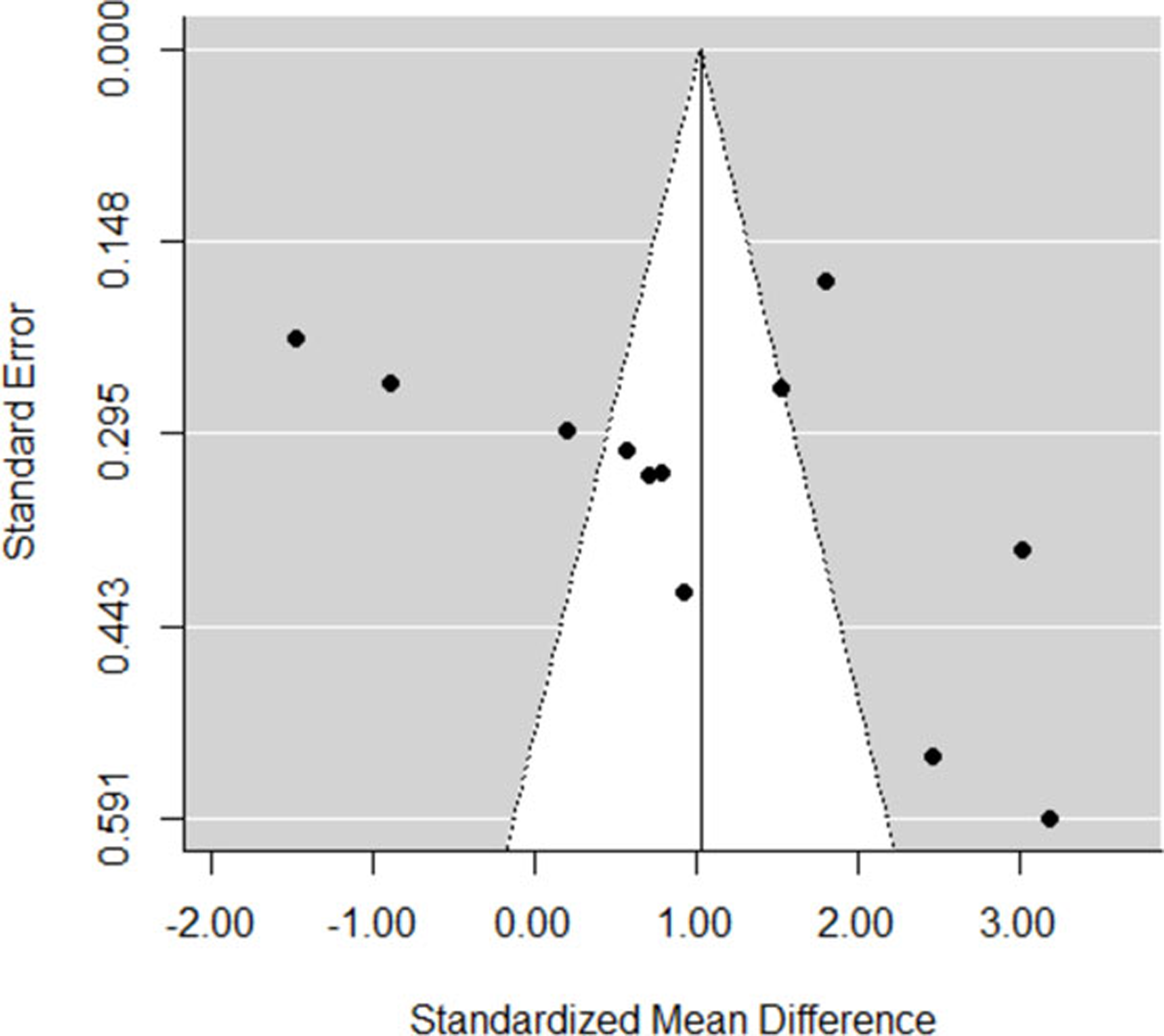

Supplement: Supplementary Figure 5 [file mp2017205x15.tif]

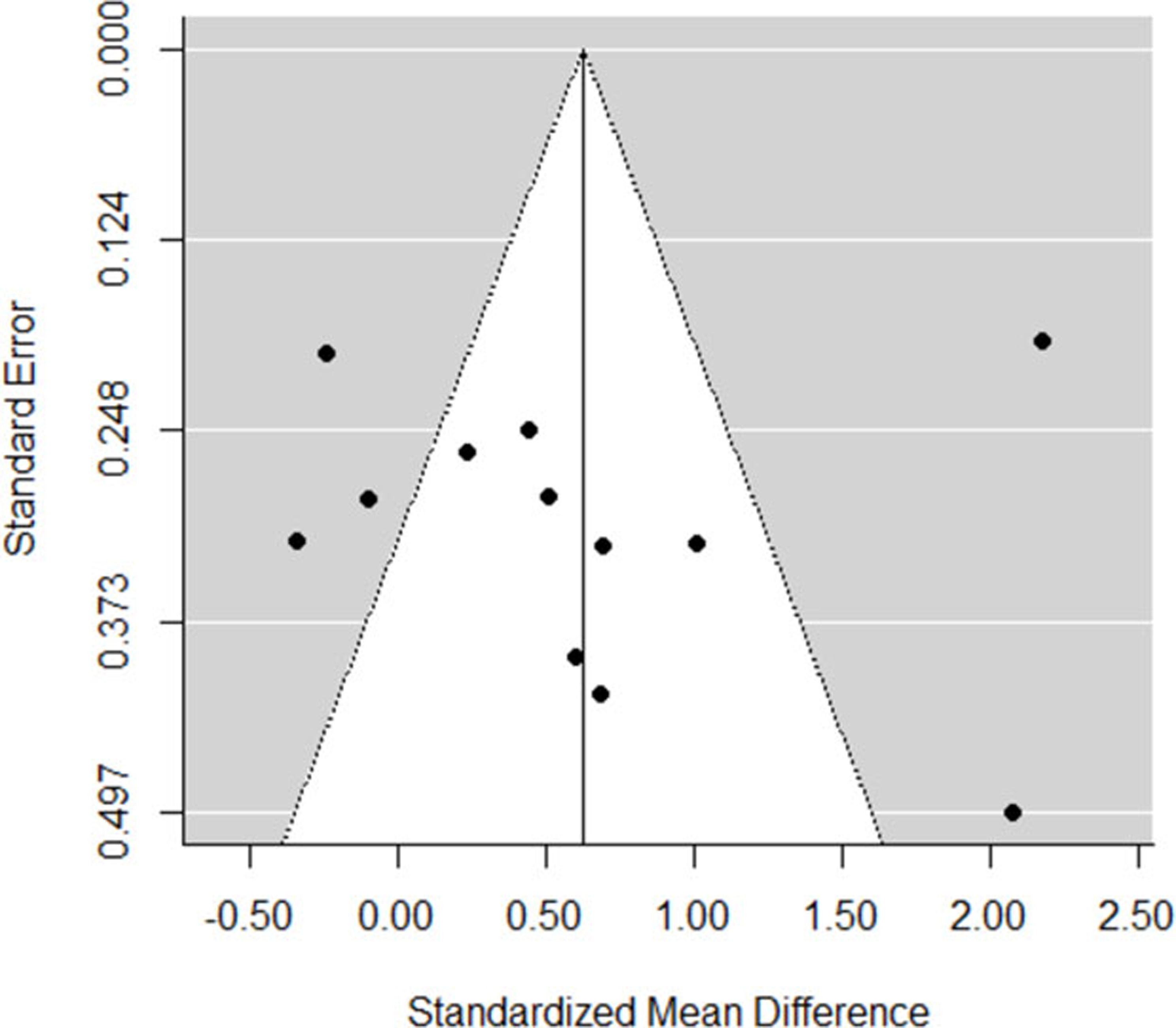

Supplement: Supplementary Figure 6 [file mp2017205x16.tif]

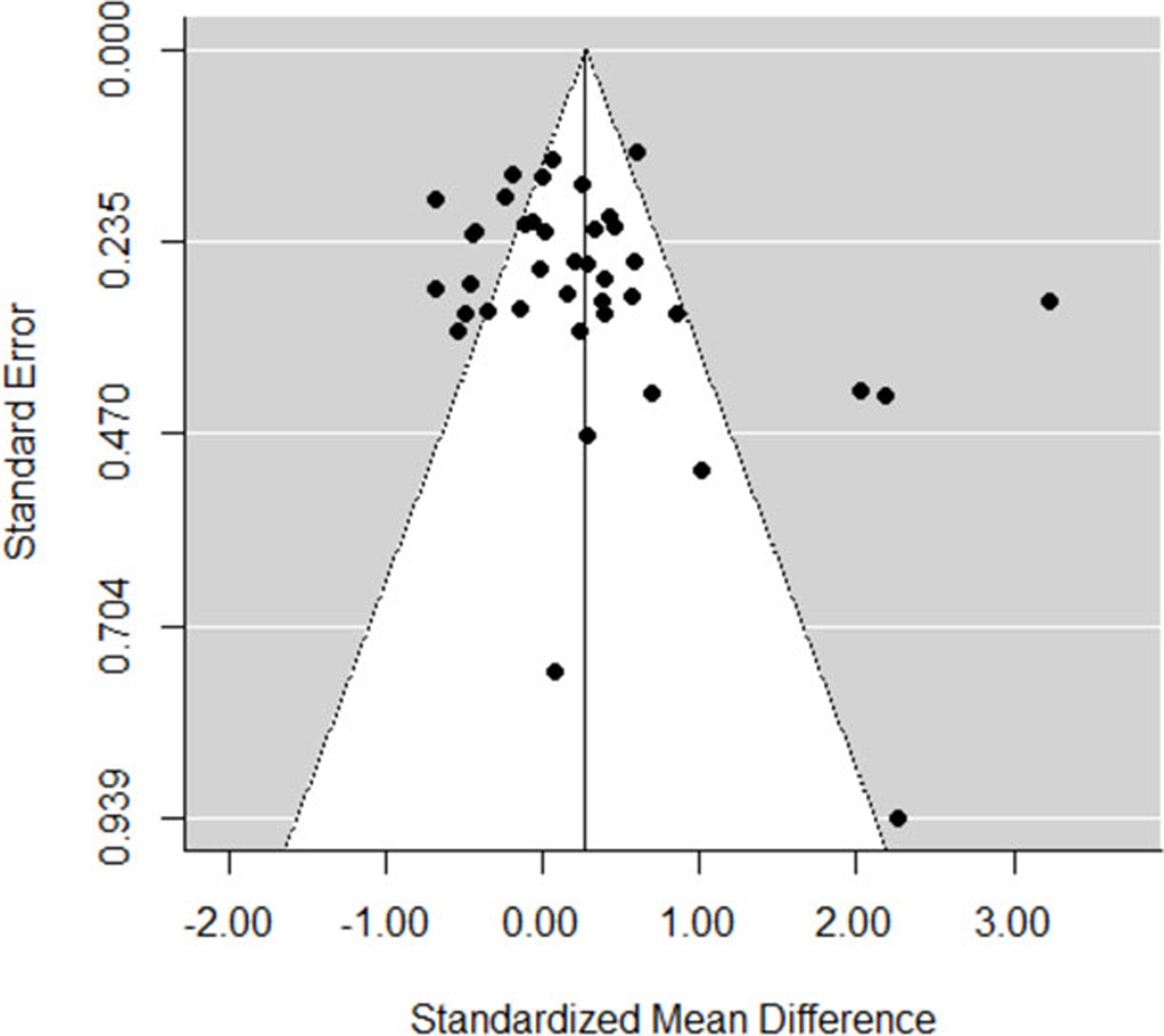

Supplement: Supplementary Figure 7 [file mp2017205x17.tif]
