# Supplementary material for: Chemokines in depression in health and in inflammatory illness: a systematic review and meta-analysis
Source: Mol Psychiatry. 2017 Nov 14;23(1):48–58. doi: 10.1038/mp.2017.205 (PMC5754468; doi:10.1038/mp.2017.205)
Supplement: Supplementary Figure Legends [file mp2017205x18.doc]

**Supplementary Figure Legends**

Supplementary Figure 1. Funnel plot of CCL2 studies of chemokine levels in plasma and serum of depressed and not depressed patients.

Supplementary Figure 2. Funnel plot of CCL3 studies of chemokine levels in plasma and serum of depressed and not depressed patients.

Supplementary Figure 3. Funnel plot of CCL4 studies of chemokine levels in plasma and serum of depressed and not depressed patients.

Supplementary Figure 4. Funnel plot of CCL11 studies of chemokine levels in plasma and serum of depressed and not depressed patients.

Supplementary Figure 5. Funnel plot of CXCL4 studies of chemokine levels in plasma and serum of depressed and not depressed patients.

Supplementary Figure 6. Funnel plot of CXCL7 studies of chemokine levels in plasma and serum of depressed and not depressed patients.

Supplementary Figure 7. Funnel plot of CXCL8 studies of chemokine levels in plasma and serum of depressed and not depressed patients.
